# Sex Differences in Behavioral Responding and Dopamine Release during Pavlovian Learning

**DOI:** 10.1523/ENEURO.0050-22.2022

**Published:** 2022-03-21

**Authors:** Merridee J. Lefner, Mariana I. Dejeux, Matthew J. Wanat

**Affiliations:** Neurosciences Institute and the Department of Neuroscience, Developmental and Regenerative Biology, The University of Texas at San Antonio, San Antonio, Texas 78249

**Keywords:** dopamine, learning, sex differences

## Abstract

Learning associations between cues and rewards require the mesolimbic dopamine system. The dopamine response to cues signals differences in reward value in well trained animals. However, these value-related dopamine responses are absent during early training sessions when cues signal differences in the reward rate. These findings suggest cue-evoked dopamine release conveys differences between outcomes only after extensive training, though it is unclear whether this is unique to when cues signal differences in reward rate, or whether this is also evident when cues signal differences in other value-related parameters such as reward size. To address this, we used a Pavlovian conditioning task in which one audio cue was associated with a small reward (one pellet) and another audio cue was associated with a large reward (three pellets). We performed fast-scan cyclic voltammetry to record changes in dopamine release in the nucleus accumbens of male and female rats throughout learning. While female rats exhibited higher levels of conditioned responding, a faster latency to respond, and elevated post-reward head entries relative to male rats, there were no sex differences in the dopamine response to cues. Multiple training sessions were required before cue-evoked dopamine release signaled differences in reward size. Reward-evoked dopamine release scaled with reward size, though females displayed lower reward-evoked dopamine responses relative to males. Conditioned responding related to the decrease in the peak reward-evoked dopamine response and not to cue-evoked dopamine release. Collectively, these data illustrate sex differences in behavioral responding as well as in reward-evoked dopamine release during Pavlovian learning.

## Visual Abstract

**Figure:**
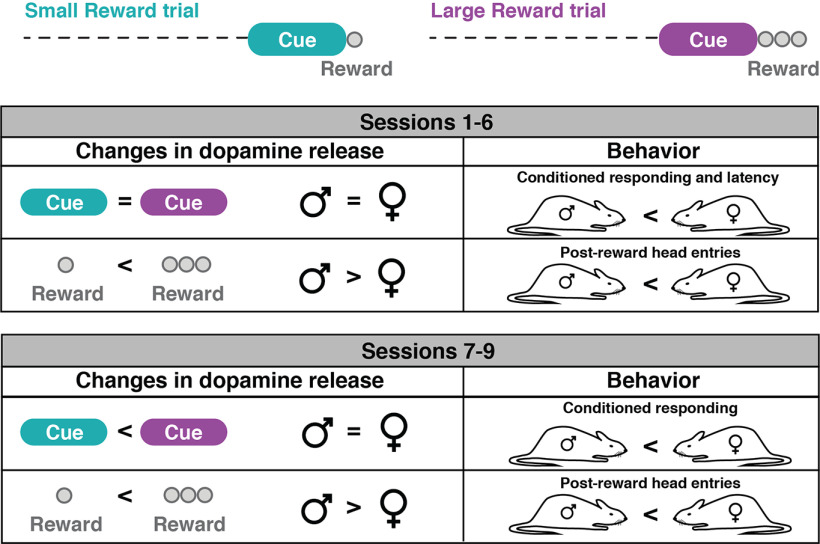

## Significance Statement

The dopamine response to cues signals differences in reward value after extensive training. However, recent studies suggest that this value encoding is not present during early sessions. We used fast-scan cyclic voltammetry to record changes in dopamine release in rats throughout pavlovian learning. We demonstrate that in the first six training sessions, cue-evoked dopamine release did not encode reward size, while reward-evoked dopamine release did. With additional training, cue-evoked dopamine release signaled differences in reward size. We identified sex differences in behavior as females displayed augmented responding during the cue and following reward delivery relative to males. Furthermore, we found sex differences in dopamine release as females exhibited a smaller reward-evoked dopamine response compared with males.

## Introduction

Efficient reward seeking involves identifying cues that predict rewards and discriminating between cues that signal different reward options. The mesolimbic dopamine system plays an integral role in regulating behavioral responses toward reward-associated cues (Phillips et al., 2007; Salamone and Correa, 2012). Cue-evoked dopamine responses convey reward-related information such as the relative reward size (Tobler et al., 2005; Roesch et al., 2007; Gan et al., 2010), reward probability (Fiorillo et al., 2003; Hart et al., 2015), and reward rate (Fonzi et al., 2017). While this effect is evident in extensively trained animals, the emergence of these signals during initial training sessions has not been well characterized. We recently used a Pavlovian conditioning task to demonstrate that cue-evoked dopamine release encodes reward rate (i.e., the time elapsed since the previous reward delivery) after extensive training (>24 sessions; Fonzi et al., 2017), but not during the first 6 training sessions (Stelly et al., 2021). These findings suggest that cue-evoked dopamine encodes reward rate through a multistep process, by first signaling an upcoming reward independent of value during initial training sessions and, after additional training, conveying the relative difference in value between cues. However, it remains unclear whether extensive training is similarly required for cue-evoked dopamine signals to convey prospective value-related parameters, such as reward size.

The aforementioned research was primarily performed in male subjects; however, increasing evidence highlights sex differences in behavioral responding. Across studies, female subjects display augmented behavioral responses compared with males, including higher locomotor activity, faster latency, and elevated conditioned responding (Lynch and Carroll, 1999; Dubroqua et al., 2011; Eubig et al., 2014; Scholl et al., 2019; Stringfield et al., 2019; Zachry et al., 2019; Kutlu et al., 2020; Bishnoi et al., 2021; Chen et al., 2021; George et al., 2021; Knight et al., 2021). Furthermore, prior research has identified sex differences in dopamine neuron activity and release (Xiao and Becker, 1994; Walker et al., 2000; McArthur et al., 2007; Becker and Chartoff, 2019; Brundage et al., 2021; Zachry et al., 2021). These differences in dopamine transmission between males and females could account for the observed sex differences in dopamine-dependent behaviors (Lynch and Carroll, 1999; Dubroqua et al., 2011; Eubig et al., 2014; Pitchers et al., 2015; Stringfield et al., 2019; Zachry et al., 2019; Kutlu et al., 2020; Chen et al., 2021; George et al., 2021). However, it is not known whether sex differences during Pavlovian learning are accompanied by distinct patterns of dopamine signaling.

In this study, we trained male and female rats on a Pavlovian task where one cue was associated with a small reward (one pellet) and another cue was associated with a large reward (three pellets). Female rats displayed higher levels of conditioned responding, a faster latency to the food port, and a higher number of post-reward head entries compared with male rats. We used fast-scan cyclic voltammetry to record changes in dopamine release in the nucleus accumbens (NAc) throughout learning. The cue-evoked dopamine response did not encode differences in reward size during the first six training sessions but did signal differences in value during later sessions. There were no differences in cue-evoked dopamine release between males and females. In contrast, the dynamics of reward-evoked dopamine release was influenced by both reward size and sex. Both male and female rats displayed higher reward-evoked dopamine release to the larger reward option, though females exhibited lower reward-evoked dopamine levels compared with males. These data illustrate that sex differences in dopamine transmission are stimulus specific.

## Materials and Methods

### Subjects and surgery

All procedures were approved by the Institutional Animal Care and Use Committee at The University of Texas at San Antonio. Male (weight range, 300–350 g) and female (weight range, 200–250 g) Sprague Dawley rats [Charles River; age range, postnatal day 60 (P60) to P65] were pair housed on arrival, and were given *ad libitum* access to water and chow and maintained on a 12 h light/dark cycle (*n* = 8 male rats/9 electrodes; 5 female rats/5 electrodes). Carbon fiber voltammetry electrodes consisted of a carbon fiber housed in silica tubing and cut to a length of ∼150 μm (Clark et al., 2010). Voltammetry electrodes were surgically implanted to target the NAc (relative to bregma: 1.3 mm anterior; ±1.3 mm lateral; 7.0 mm ventral) along with an Ag/AgCl reference electrode. Rats were single housed following surgery and allowed to recover for >3 weeks before beginning training.

### Behavioral procedures

After recovering from surgery, rats were placed and maintained on mild food restriction (standard laboratory chow: ∼8 g/d for females; ∼15 g/d for males) to target 90% free-feeding weight, allowing for an increase of 1.5% per week. Behavioral sessions were performed in chambers (Med Associates) that had grid floors, a house light, a food tray, and auditory stimulus generators (2.5 and 4.5 kHz tones). The chamber floors were thoroughly cleansed with a disinfectant, and the walls and food port were cleaned with 70% ethanol solution between every subject. To familiarize rats with the chamber and food retrieval, rats underwent a single magazine training session in which 20 food pellets (45 mg; BioServ) were noncontingently delivered at a 90 ± 15 s variable interval. Rats then underwent up to nine Pavlovian conditioning sessions (1/d) that each consisted of 50 trials where the termination of a 5 s audio cue [conditioned stimulus (CS); 2.5 or 4.5 kHz tone, counterbalanced across animals] resulted in the delivery of a single food pellet (Small Reward trials) or three food pellets (Large Reward trials) and illumination of the food port light for 4.5 s. The three food pellets on Large Reward trials were delivered within 0.4 s after the end of the CS presentation. Each session contained 25 Small Reward trials and 25 Large Reward trials delivered in a pseudorandom order, with a 45 ± 5 s intertrial interval between all trials. Conditioned responding was quantified as the change in the rate of head entries during the 5 s CS relative to the 5 s preceding the CS delivery (Fonzi et al., 2017; Stelly et al., 2021). We also quantified the latency to initiate a head entry during the CS. For the post-unconditioned stimulus (US) analysis, we calculated the average number of head entries made during a 9 s post-US delivery time window. For more detailed analysis, we also broke the 9 s time window into two 4.5 s epochs that corresponded to when the food tray light was illuminated (Early US; 0–4.5 s) and an equivalent period of time when the food tray light was turned off (Late US; 4.5–9 s).

### Voltammetry recordings and analysis

Chronically implanted electrodes were connected to a head-mounted amplifier to monitor changes in dopamine release in behaving rats using fast-scan cyclic voltammetry, as described previously (Clark et al., 2010; Fonzi et al., 2017; Oliva and Wanat, 2019; Stelly et al., 2019, 2020, 2021; Oliva et al., 2021). The carbon fiber electrodes were held at −0.4 V (vs Ag/AgCl) with voltammetric scans applied at 10 Hz in which the potential was ramped in a triangular waveform to +1.3 V and back to −0.4 V at a rate of 400 V/s. A principal component regression analysis (Heien et al., 2005) was performed on the voltammetry signal using a standard training set that accounts for dopamine, pH, and drift. The average postimplantation sensitivity of electrodes (34 nA/μM) was used to estimate dopamine concentration (Clark et al., 2010). Chemical verification of dopamine was achieved by obtaining a high correlation of the cyclic voltammogram during a reward-related event to that of a dopamine standard (correlation coefficient *r*^2^ ≥ 0.75, by linear regression). Voltammetry data for a session were excluded from analysis if the detected voltammetry signal did not satisfy these chemical verification criteria (Fonzi et al., 2017; Stelly et al., 2021). Voltammetry data for a given trial were excluded if the principal component regression analysis failed to extract dopamine current on >25% of the data points for a given trial (i.e., the residual Q value for the regression analysis exceeded the 95.5% confidence limit for the training set; Heien et al., 2005; Rodeberg et al., 2017; Stelly et al., 2020, 2021).

The CS-evoked dopamine response was quantified as the average dopamine response during the 5 s CS relative to the 5 s before the CS delivery (Fonzi et al., 2017; Stelly et al., 2021). The peak US-evoked dopamine response was quantified as the maximum dopamine response in the 3 s following US delivery relative to the 0.5 s before US delivery. The area under the curve (AUC) of the post-US dopamine response was quantified as the average dopamine response in the 9 s following US delivery relative to 0.5 s before US delivery. To determine potential differences in the decay of reward-evoked dopamine release between males and females, we normalized and aligned to the peak US dopamine response following the Small Reward delivery of the first session. These data were then fit to a single-phase decay curve to calculate the tau for each electrode (Oliva and Wanat, 2019).

### Data analysis

Statistical analyses were performed in GraphPad Prism 9 and RStudio. Behavioral responding and dopamine quantification were analyzed using a mixed-effects model fit (restricted maximum likelihood method), repeated measures (rm) where appropriate, followed by a *post hoc* Sidak’s test. The Geisser–Greenhouse correction was applied to address unequal variances between groups. A repeated-measures correlation was used to correlate dopamine signals and behavioral outcomes across all subjects, trial types, and training sessions (Bakdash and Marusich, 2017; Stelly et al., 2021). The full list of statistical analyses is presented in Extended Data Tables 1-1, 2-1, 3-1, 4-1, and 5-1.

10.1523/ENEURO.0050-22.2022.t1-1 Extended Data Table 1-1 Panel B - Conditioned responding. Download Table 1-1, DOC file.

10.1523/ENEURO.0050-22.2022.t2-1 Extended Data Table 2-1 Panel B -Post US head entries. Download Table 2-1, DOC file.

10.1523/ENEURO.0050-22.2022.t3-1 Extended Data Table 3-1 Panel D - CS-evoked dopamine release. Download Table 3-1, DOC file.

10.1523/ENEURO.0050-22.2022.t4-1 Extended Data Table 4-1 Panel B - CS-evoked dopamine: Sessions 7-9. Download Table 4-1, DOC file.

10.1523/ENEURO.0050-22.2022.t5-1 Extended Data Table 5-1 Sessions 1-9 Repeated measures correlation. Download Table 5-1, DOC file.

### Histology

Rats were deeply anesthetized, and electrical lesions were applied to the voltammetry electrodes followed by intracardial perfusion with 4% paraformaldehyde. Brains were removed and postfixed for at least 24 h, then subsequently placed in 15% and 30% sucrose solutions in PBS. Brains were then flash frozen on dry ice, coronally sectioned, and stained with cresyl violet. Electrode locations were mapped onto a standardized rat brain atlas.

## Results

**Figure 1.**
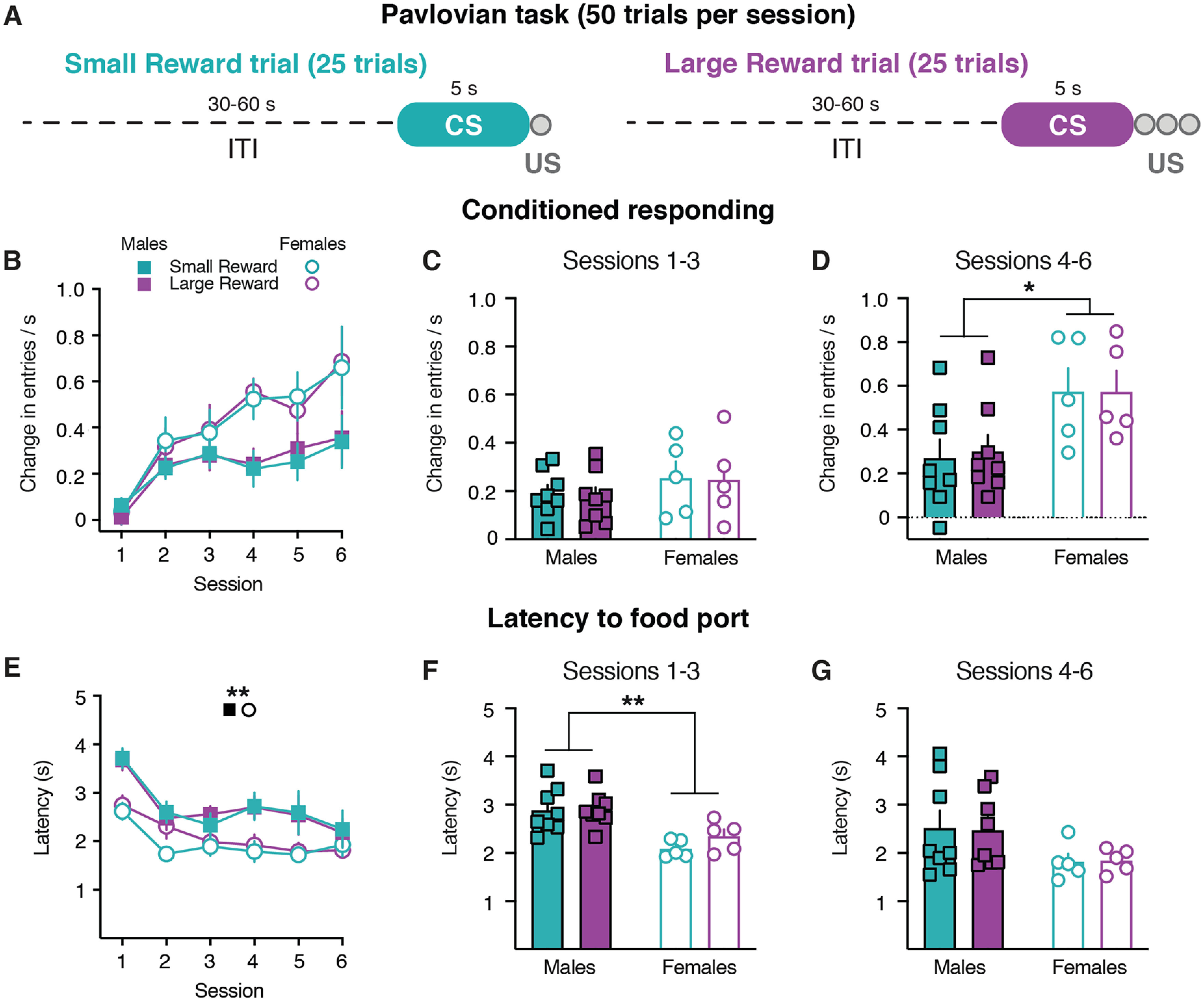
Sex differences in behavioral responding during CS presentation. ***A***, Training schematic for the Pavlovian reward size task. ***B***, Conditioned responding for males (filled squares) and females (open circles) during Small Reward (teal) and Large Reward (purple) trials. ***C***, Conditioned responding averaged across the first three sessions of training. ***D***, Conditioned responding averaged across the latter three sessions of training. ***E***, Latency to respond to the food port. ***F***, Latency to respond averaged across the first three sessions of training. ***G***, Latency to respond averaged across the latter three sessions of training. *p <* *0.05, **p <* *0.01.

Rats were trained on a Pavlovian conditioning task in which one audio cue (CS) signaled the delivery of a single sucrose pellet (US; Small Reward trial) and another audio cue signaled the delivery of three sucrose pellets (Large Reward trial, Fig. 1*A*). Conditioned responding was quantified as the change in the rate of head entries during the 5 s CS relative to the rate of head entries during the 5 s preceding the CS (Fonzi et al., 2017; Stelly et al., 2020, 2021). Rats increased conditioned responding across sessions, with no difference between Small and Large Reward cues (three-way mixed-effects analysis; session effect: *F*_(2.26,24.86)_ = 14.01, *p *<* *0.0001; reward size effect: *F*_(1,11)_ = 0.03, *p *=* *0.86; *n *=* *13 rats; Fig. 1*B*, Extended Data Table 1-1). There was a trend for enhanced conditioned responding in female rats (sex effect: *F*_(1,55)_ = 3.90, *p *=* *0.05; session × sex interaction: *F*_(5,55)_ = 2.34, *p *=* *0.05; Fig. 1*B*, Extended Data Table 1-1). Rats also decreased the latency to the food port across training sessions, with no difference between Small and Large reward trials (three-way mixed-effects analysis; session effect: *F*_(2.94,32.33)_ = 6.26, *p *<* *0.002; reward size effect: *F*_(1,11)_ = 0.51, *p *=* *0.49; Fig. 1*E*, Extended Data Table 1-1). Females displayed a faster latency to respond across sessions compared with males (sex effect: *F*_(1,55)_ = 8.80, *p *=* *0.004; Fig. 1*E*, Extended Data Table 1-1), consistent with prior findings (Eubig et al., 2014; Stringfield et al., 2019). We further analyzed these behavioral responses when averaged into three-session bins. During the first three sessions there were no sex differences in conditioned responding (two-way mixed-effects analysis; sex effect: *F*_(1,11)_ = 0.90, *p *=* *0.36; Fig. 1*C*, Extended Data Table 1-1), though females exhibited a faster latency to enter the food port (two-way mixed-effects analysis; sex effect: *F*_(1,11)_ = 14.56, *p *=* *0.003; Fig. 1*F*, Extended Data Table 1-1). During the latter three sessions, female rats displayed higher levels of conditioned responding (two-way mixed-effects analysis; sex effect: *F*_(1,11)_ = 5.11, *p *<* *0.05; Fig. 1*D*, Extended Data Table 1-1), though there were no sex differences in the latency to respond (two-way mixed-effects analysis; sex effect: *F*_(1,11)_ = 2.71, *p *=* *0.13; Fig. 1*G*, Extended Data Table 1-1). Collectively, these findings illustrate that female rats display augmented behavioral responding within the CS presentation compared with male rats during the first six training sessions of Pavlovian learning. However, these behavioral responses during the cue presentation did not reflect differences in the upcoming reward size.

**Figure 2.**
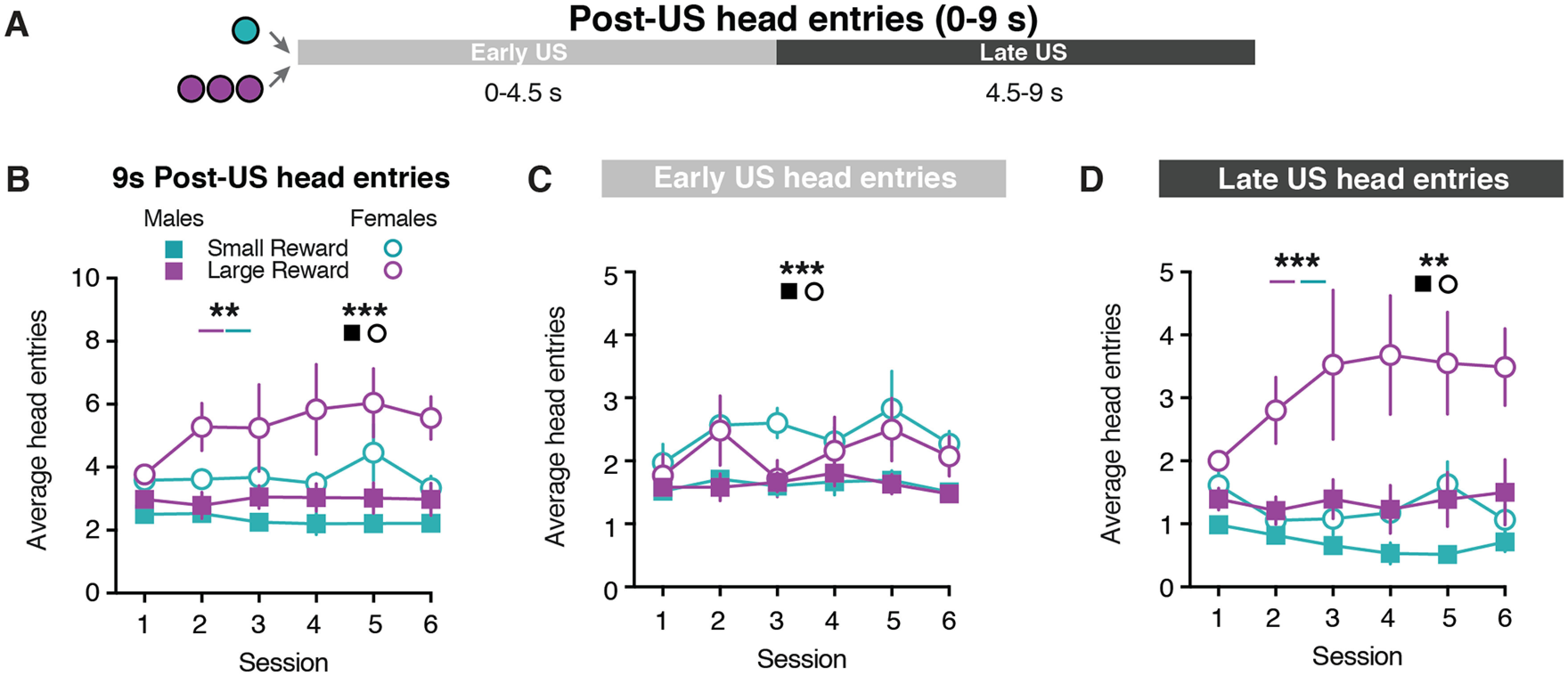
Sex differences in behavioral responding during US presentation. ***A***, Schematic for post-US epochs: Early US (0–4.5 s); Late US (4.5–9 s). ***B***, Average head entries made during the full 9 s post-US window for Small Reward and Large Reward trials. ***C***, Average head entries made during the Early US for Small Reward and Large Reward trials. ***D***, Average head entries made during the Late US for Small Reward and Large Reward trials. **p <* *0.01, ***p <* *0.001.

Given the sex differences in CS-evoked behavior, we next examined whether male and female rats differed in their behavioral responses following the reward delivery. Female rats performed a higher number of non-CS head entries relative to males (Extended Data Fig. 1-1), which suggests that females performed more head entries following the US. To address this possibility, we examined the head entries performed in the 9 s after the reward was delivered (Fig. 2*A*). Female rats exhibited greater post-US head entries compared with male rats (sex effect: *F*_(1,55)_ = 17.44, *p *=* *0.0001; Fig. 2*B*, Extended Data Table 2-1). Additionally, rats performed more head entries following the delivery of the Large Reward (three-way mixed-effects analysis; reward size effect: *F*_(1,11)_ = 10.15, *p *=* *0.009; Fig. 2*B*, Extended Data Fig. 2-1 and Table 2-1). We further examined the post-US head entries in two separate epochs that corresponded to when the food tray light was illuminated (Early US: 0–4.5 s) and an equivalent period of time when the food tray light was turned off (Late US: 4.5–9 s; Fig. 2*A*). During the Early US epoch, female rats made a greater number of head entries compared with male rats (three-way mixed-effects analysis; sex effect: *F*_(1,55)_ = 13.60, *p *=* *0.0005; Fig. 2*C*, Extended Data Fig. 2-1 and Table 2-1). During the Late US epoch, rats performed more head entries following the Large Reward delivery (three-way mixed-effects analysis; reward size effect: *F*_(1,11)_ = 24.65, *p *=* *0.0004; Fig. 2*D*, Extended Data Fig. 2-1 and Table 2-1). Furthermore, there was a sex × reward size interaction effect as female rats continued to demonstrate a greater number of head entries than males throughout the Late US epoch (sex effect: *F*_(1,55)_ = 12.20, *p *=* *0.001; sex × reward size effect: *F*_(1,55)_ = 5.94, *p *=* *0.02; Fig. 2*D*, Extended Data Fig. 2-1 and Table 2-1). Together, these results illustrate that sex and reward size influence the number of post-US head entries.

10.1523/ENEURO.0050-22.2022.f1-1 Figure 1-1 Number of head entries across sessions. ***A***, Total number of head entries across sessions in males (black square) and females (open circle; two-way mixed-effects analysis; session effect: *F*_(5,55)_ = 1.98, *p* = 0.10; sex effect: *F*_(1,11)_ = 8.38, *p* = 0.02; interaction effect: *F*_(5,55)_ = 1.25, *p* = 0.30). ***B***, CS head entries across sessions (two-way mixed-effects analysis; session effect: *F*_(2.25,26.69)_ = 15.34, p < 0.0001; sex effect: *F*_(1,11)_ = 12.10, *p* = 0.005; interaction effect: *F*_(5,55)_ = 0.97, *p* = 0.44). ***C***, Non-CS head entries across sessions (two-way mixed-effects analysis; session effect: *F*_(2.47,27.11)_ = 0.73, *p* = 0.52; sex effect: *F*_(1,11)_ = 6.12, *p* = 0.03; interaction effect: *F*_(5,55)_ = 1.59, *p* = 0.18). *p <* *0.05, **p <* *0.01. Download Figure 1-1, TIF file.

10.1523/ENEURO.0050-22.2022.f2-1 Figure 2-1 Sex differences in behavioral responding during US presentation. ***A***, Head entries during the 9 s postreward window averaged across the first three sessions of training (two-way mixed-effects analysis; reward size effect: *F*_(1,11)_ = 15.03, *p* = 0.003; sex effect: *F*_(1,11)_ = 11.27, *p* = 0.006; interaction effect: *F*_(1,11)_ = 2.17, *p* = 0.17; *post hoc* Sidak’s test—sex: Small Reward: *t*_(22)_ = 2.41, *p* < 0.05; Large Reward: *t*_(22)_ = 3.67, *p* = 0.003; *post hoc* Sidak’s test—reward size: males: *t*_(11)_ = 1.94, *p* = 0.15; females: *t*_(11)_ = 3.41, *p* = 0.01). ***B***, Head entries during the 9 s postreward window averaged across the latter three sessions of training (two-way mixed-effects analysis; reward size effect: *F*_(1,22)_ = 7.48, *p* = 0.01; sex effect: *F*_(1,22)_ = 17.45, *p* = 0.0004; interaction effect: *F*_(1,22)_ = 1.44, *p* = 0.24; *post hoc* Sidak’s test—sex; Small Reward: *t*_(22)_ = 2.11, *p* = 0.09; Large Reward: *t*_(22)_ = 3.80, *p* = 0.002; *post hoc* Sidak’s test—reward size; males: *t*_(22)_ = 1.24, *p* = 0.41; females: *t*_(22)_ = 2.51, *p* = 0.04). ***C***, Head entries during the Early US averaged across the first three sessions of training (two-way mixed-effects analysis; reward size effect: *F*_(1,11)_ = 5.47, *p* = 0.04; sex effect: *F*_(1,11)_ = 8.45, *p* = 0.01; interaction effect: *F*_(1,11)_ = 5.29, *p* = 0.04; *post hoc* Sidak’s test—sex: Small Reward: *t*_(22)_ = 3.57, *p* = 0.003; Large Reward: *t*_(22)_ = 1.78, *p* = 0.17; *post hoc* Sidak’s test—reward size; males: *t*_(11)_ = 0.03, *p* = 0.99; females: *t*_(11)_ = 2.96, *p* = 0.03). ***D***, Head entries during the Early US averaged across the latter three sessions of training (two-way mixed-effects analysis; reward size effect: *F*_(1,11)_ = 0.27, *p* = 0.61; sex effect: *F*_(1,11)_ = 9.67, *p* = 0.01; interaction effect: *F*_(1,11)_ = 0.35, *p* = 0.57; *post hoc* Sidak’s test—sex; Small Reward: *t*_(22)_ = 2.72, *p* = 0.02; Large Reward: *t*_(22)_ = 1.94, *p* = 0.13; *post hoc* Sidak’s test—reward size; males: *t*_(11)_ = 0.71, *p* = 0.99; females: *t*_(11)_ = 0.71, *p* = 0.74). ***E***, Head entries during the Late US averaged across the first three sessions of training (two-way mixed-effects analysis; reward size effect: *F*_(1,11)_ = 27.85, *p* = 0.0003; sex effect: *F*_(1,11)_ = 10.08, *p* = 0.009; interaction effect: *F*_(1,11)_ = 6.86, *p* = 0.02; *post hoc* Sidak’s test—sex; Small Reward: *t*_(22)_ = 1.22, *p* = 0.41; Large Reward: *t*_(22)_ = 4.09, *p* = 0.001; *post hoc* Sidak’s test—reward size; males: *t*_(11)_ = 2.14, *p* = 0.11; females: *t*_(11)_ = 5.03, *p* = 0.0008). ***F***, Head entries during the Late US averaged across the latter three sessions of training (two-way mixed-effects analysis; reward size effect: *F*_(1,11)_ = 17.75, *p* = 0.002; sex effect: *F*_(1,11)_ = 11.61, *p* = 0.006; interaction effect: *F*_(1,11)_ = 4.22, *p* = 0.06; *post hoc* Sidak’s test—sex: small reward: *t*_(22)_ = 1.26, *p* = 0.40; large reward: *t*_(22)_ = 3.92, *p* = 0.002; *post hoc* Sidak’s test—reward size; males: *t*_(11)_ = 1.74, *p* = 0.21; females: *t*_(11)_ = 3.99, *p* = 0.004). *Indicates the main effect of sex or the *post hoc* effect of sex: *p <* *0.05, **p <* *0.01, ***p <* *0.001. #Indicates the *post hoc* effect of reward size: ##*p *<* *0.01, ###*p *<* *0.001. Download Figure 2-1, TIF file.

**Figure 3.**
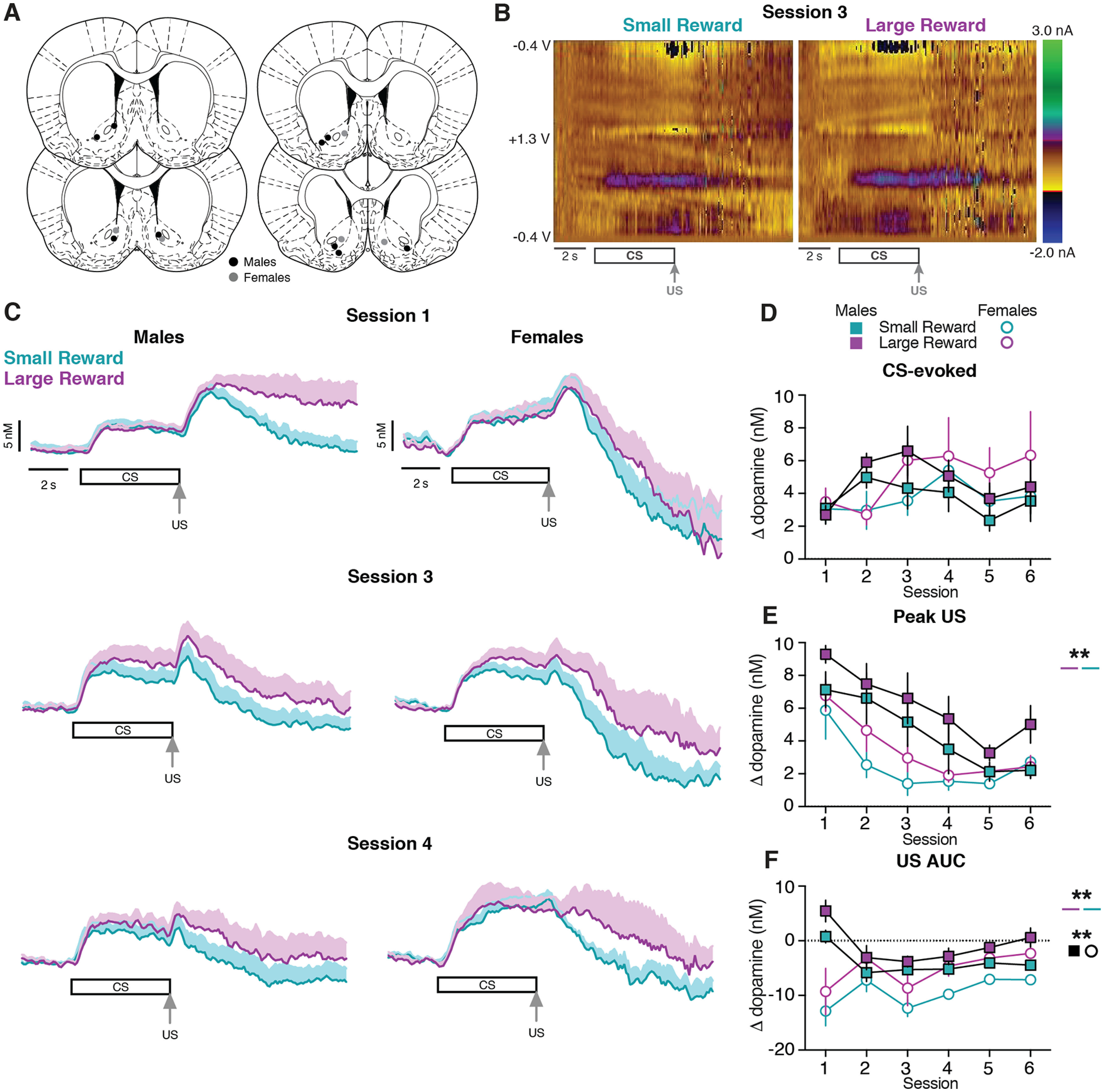
Dopamine release in the NAc during early training sessions. ***A***, Location of voltammetry electrodes in males (black) and females (gray). ***B***, Representative two-dimensional pseudocolor plots of the resulting current from voltage sweeps (*y*-axis) as a function of time (*x*-axis) of voltammetry recordings in the NAc. ***C***, Average dopamine signals across training sessions in males (left) and females (right). ***D***, Average CS-evoked dopamine release across sessions. ***E***, Average Peak US-evoked dopamine release across sessions. ***F***, Average US AUC-evoked dopamine release across sessions. **p <* *0.01.

The emergence of Pavlovian conditioned responses depends on dopamine signaling within the ventral striatum (Darvas et al., 2014). Here, we performed voltammetry recordings in the NAc to examine how the CS- and US-evoked dopamine responses progressed across training (Fig. 3*A*,*B*). Both male and female subjects exhibited dopamine release to the CS presentation (Fig. 3*C*). We quantified CS-evoked dopamine release as the average response during the 5 s CS relative to the 5 s before the CS, identical to the manner in which conditioned responding was calculated (Fig. 1). CS-evoked dopamine release did not differ between sexes or trial type in the first six training sessions (three-way mixed-effects analysis; session effect: *F*_(1.97,23.64)_ = 3.22, *p = *0.06; sex effect: *F*_(1,30)_ = 0.07, *p *=* *0.80; reward size effect: *F*_(1,12)_ = 3.54, *p *=* *0.09; Fig. 3*D*, Extended Data Table 3-1).

To determine whether reward-evoked dopamine release scaled with reward size, we quantified the maximum dopamine response during the 3 s after the reward was delivered relative to the 0.5 s before the US delivery (peak US). Both male and female rats display a higher peak US dopamine response during Large Reward trials compared with Small Reward trials (three-way mixed-effects analysis; reward size effect: *F*_(1,12)_ = 17.40, *p = *0.001; Fig. 3*C*,*E*, Extended Data Table 3-1). We additionally analyzed the area under the curve for the average dopamine response during the 9 s after the US delivery relative to 0.5 s at the end of the CS (US AUC). Dopamine levels during this post-US period were higher following delivery of the Large Reward compared with the Small Reward (three-way mixed-effects analysis; reward size effect: *F*_(1,12)_ = 17.98, *p *=* *0.001; Fig. 3*F*, Extended Data Table 3-1). Furthermore, female rats displayed a lower US AUC dopamine response compared with male rats (three-way mixed-effects analysis; sex effect: *F*_(1,30)_ = 7.91, *p *=* *0.009; Fig. 3*F*, Extended Data Table 3-1). This difference in dopamine levels between trial types was also evident when examining the dopamine response during the Early and Late post-US epochs (Extended Data Fig. 3-1).

10.1523/ENEURO.0050-22.2022.f3-1 Figure 3-1 Post-US dopamine response in Early and Late epochs. ***A***, Average dopamine signals in first session in males (left) and females (right) depicting Early and Late epochs. ***B***, Average Early US-evoked dopamine release across sessions (three-way mixed-effects analysis; session effect: *F*_(2.77,33.19)_ = 8.43, *p* = 0.0004; sex effect: *F*_(1,30)_ = 3.68, *p* = 0.06; reward size effect: *F*_(1,12)_ = 19.10, *p* = 0.0009; session × sex effect: *F*_(5,30)_ = 4.64, *p* = 0.003; session × reward size effect: *F*_(2.38,14.28)_ = 1.18, *p* = 0.34; sex × reward size effect: *F*_(1,30)_ = 1.01, *p* = 0.32; interaction effect: *F*_(5,30)_ = 1.22, *p* = 0.33). ***C***, Average Late US-evoked dopamine release across sessions (three-way mixed-effects analysis; session effect: *F*_(1.80,21.56)_ = 2.04, *p* = 0.16; sex effect: *F*_(1,30)_ = 9.49, *p* = 0.004; reward size effect: *F*_(1,12)_ = 15.32, *p* = 0.002; session × sex effect: *F*_(5,30)_ = 6.46, *p* = 0.0003; session × reward size effect: *F*_(1.77,10.62)_ = 0.58, *p* = 0.56; sex × reward size effect: *F*_(1,30)_ = 0.01, *p* = 0.93; interaction effect: *F*_(5,30)_ = 1.62, *p* = 0.18). **p <* *0.01, ***p <* *0.001. Download Figure 3-1, TIF file.

We next examined whether the lower post-US dopamine levels in female rats could be explained by a difference in dopamine clearance. To address this, the data were normalized to the peak US dopamine response and fit to a single-phase decay curve (Oliva and Wanat, 2019). This analysis was performed on the first training session when there was a robust US dopamine response and only for the Small Reward trials to minimize the potential influence of multiple reward deliveries on the dynamics of the dopamine response. Females exhibited a decreased plateau (i.e., lower dopamine levels) compared with males (unpaired *t* test, *t*_(9)_ = 2.65, *p = *0.03; Extended Data Fig. 3-2). However, there was no difference in the tau between male and female rats (unpaired *t* test, *t*_(9)_ = 0.92, *p = *0.38; Extended Data Fig. 3-2), which indicates that the rate of the decay of the US-evoked dopamine response is not influenced by sex. Collectively, these results suggest that in contrast to CS-evoked dopamine release, US-evoked dopamine release encodes differences in reward size throughout the post-US period. Additionally, female rats exhibited a smaller US-evoked dopamine response relative to male rats.

10.1523/ENEURO.0050-22.2022.f3-2 Figure 3-2 Single-phase decay analysis on US dopamine response. ***A***, Average US-evoked dopamine signals normalized to the peak dopamine response in Small Reward trials during the first session in males and females. ***B***, Decay plateau (unpaired *t* test; *t*_(9)_ = 2.65, *p* = 0.03). ***C***, Decay rate (unpaired *t* test; *t*_(9)_ = 0.92, *p* = 0.38). *p <* *0.05. Download Figure 3-2, TIF file.

**Figure 4.**
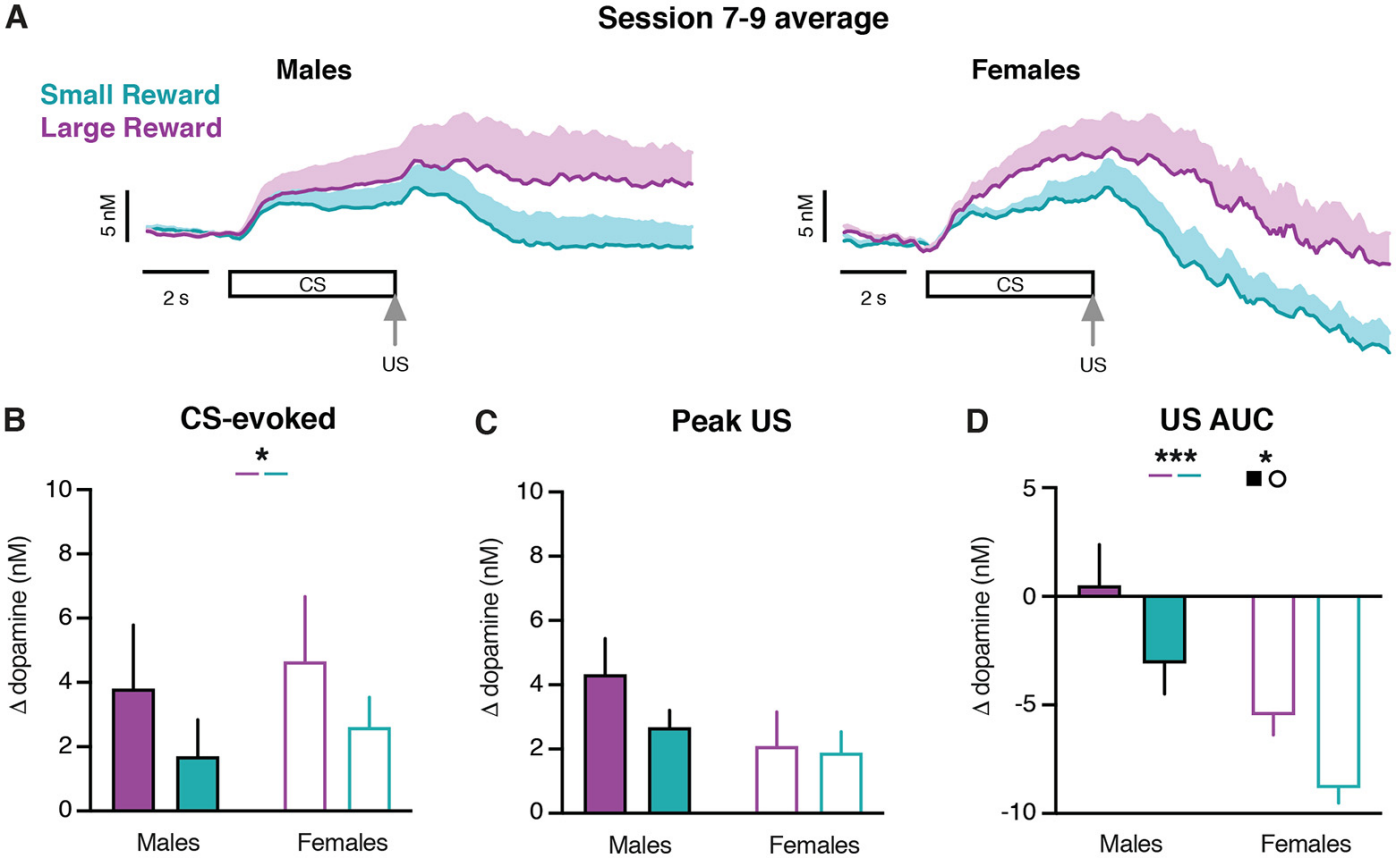
Dopamine release in the NAc during late training sessions. ***A***, Average of sessions 7–9 dopamine signals in males (left) and females (right). ***B***, Average CS-evoked dopamine release. ***C***, Average Peak US-evoked dopamine release. ***D***, Average US AUC-evoked dopamine release. *p <* *0.05, ***p <* *0.001.

CS-evoked dopamine release did not convey differences in reward size during early training sessions (Fig. 4). However, many studies demonstrate that the dopamine response to cues can convey differences in reward value in well trained animals (Fiorillo et al., 2003; Tobler et al., 2005; Roesch et al., 2007; Gan et al., 2010; Hart et al., 2015; Fonzi et al., 2017). To determine whether differences in CS-evoked dopamine emerge with further training, a subset of rats underwent three additional training sessions. In contrast to the first six training sessions, CS-evoked dopamine release signals differences in reward size in the following three training sessions (two-way mixed-effects analysis; reward size effect: *F*_(1,10)_ = 5.78, *p *=* *0.04; Fig. 4*A*,*B*, Extended Data Table 4-1). The peak US dopamine response did not differ by trial type in later sessions (two-way mixed-effects analysis; reward size effect: *F*_(1,10)_ = 2.83, *p *=* *0.12; Fig. 4*C*, Extended Data Table 4-1). However, the US AUC dopamine response remained higher following delivery of the Large Reward compared with the Small Reward (three-way mixed-effects analysis; reward size effect: *F*_(1,10)_ = 24.54, *p *=* *0.0006; Fig. 4*D*, Extended Data Table 4-1), and lower in female rats compared with male rats (three-way mixed-effects analysis; sex effect: *F*_(1,10)_ = 8.76, *p *=* *0.01; Fig. 4*D*, Extended Data Fig. 4-2), which is consistent with the findings from the first six training sessions. Furthermore, significant sex differences in conditioned responding and the post-US head entries were also evident during these later training sessions (Extended Data Fig. 4-1).

10.1523/ENEURO.0050-22.2022.f4-1 Figure 4-1 Behavioral responding during sessions 7–9. ***A***, Conditioned responding (three-way mixed-effects analysis; session effect: *F*_(1.99,21.95)_ = 0.42, *p* = 0.66; sex effect: *F*_(1,18)_ = 5.56, *p* = 0.03; reward size effect: *F*_(1,11)_ = 0.11 *p* = 0.74; session × sex effect: *F*_(2,18)_ = 0.33, *p* = 0.72; session × reward size effect: *F*_(1.76,15.87)_ = 0.23, *p* = 0.77; sex × reward size effect: *F*_(1,18)_ = 0.13, *p* = 0.72; interaction effect: *F*_(2,18)_ = 0.13, *p* = 0.88). ***B***, Latency to respond (three-way mixed-effects analysis; session effect: *F*_(1.68,18.46)_ = 1.00, *p* = 0.37; sex effect: *F*_(1,11)_ = 1.68, *p* = 0.84; reward size effect: *F*_(1,11)_ = 0.04 *p* = 0.84; session × sex effect: *F*_(2,18)_ = 0.07, *p* = 0.94; session × reward size effect: *F*_(1.83,16.43)_ = 1.61, *p* = 0.23; sex × reward size effect: *F*_(1,18)_ = 0.003, *p* = 0.95; interaction effect: *F*_(2,18)_ = 1.51, *p* = 0.25). ***C***, Post-US head entries (three-way mixed-effects analysis; session effect: *F*_(1.52,16.67)_ = 0.86, *p* = 0.41; sex effect: *F*_(1,18)_ = 14.62, *p* = 0.001; reward size effect: *F*_(1,11)_ = 5.82 *p* = 0.03; session × sex effect: *F*_(2,18)_ = 1.46, *p* = 0.26; session × reward size effect: *F*_(1.25,11.20)_ = 0.93, *p* = 0.38; sex × reward size effect: *F*_(1,18)_ = 1.93, *p* = 0.18; interaction effect: *F*_(2,18)_ = 0.17, *p* = 0.84). *p <* *0.05, **p <* *0.01. Download Figure 4-1, TIF file.

**Figure 5.**
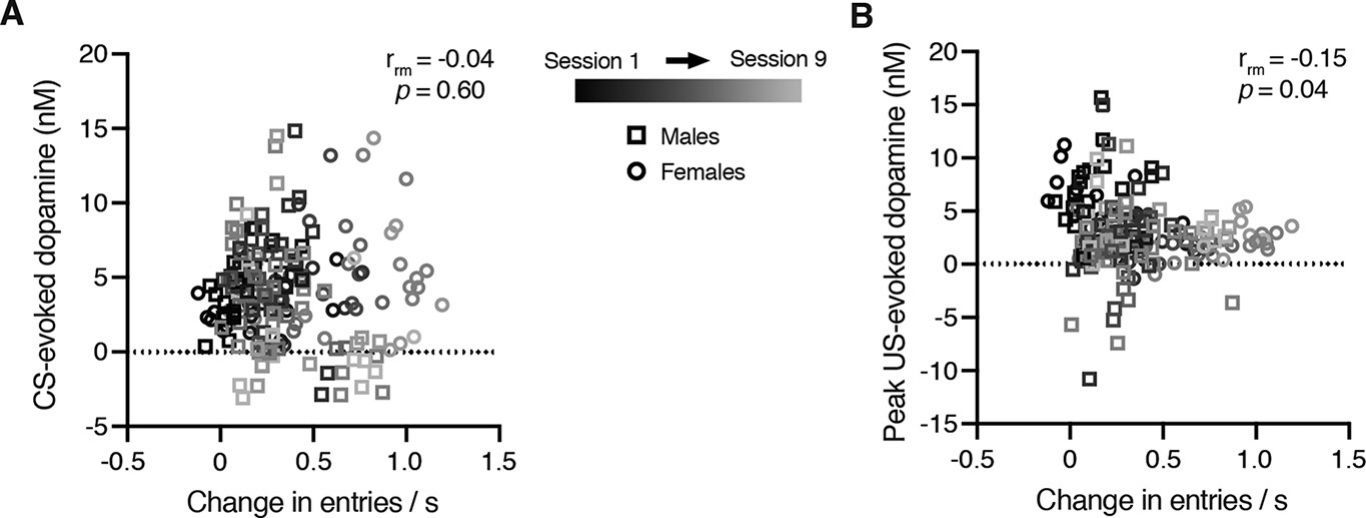
Relationship between dopamine and behavioral responding. ***A***, Relationship between CS-evoked dopamine release and conditioned responding. ***B***, Relationship between peak US-evoked dopamine release and conditioned responding.

Prior studies have linked CS- and US-evoked dopamine release to conditioned responding (Darvas et al., 2014; Fonzi et al., 2017; Coddington and Dudman, 2018; Heymann et al., 2020; Lee et al., 2020; Morrens et al., 2020; Stelly et al., 2020, 2021). Here, we used a repeated-measures correlation analysis to determine how conditioned responding relates to dopamine transmission across all subjects and training sessions. While CS-evoked dopamine release was not correlated with conditioned responding (repeated-measures correlation; conditioned responding: *r*_rm_ = −0.04, *p *=* *0.60; latency: *r*_rm_ = 0.06, *p *=* *0.40; Fig. 5*A*, Extended Data Table 5-1), there was an inverse relationship between conditioned responding and the peak US dopamine response (repeated-measures correlation: *r*_rm_ = −0.15, *p *=* *0.04; Fig. 5*B*, Extended Data Table 5-1). Furthermore, the number of head entries occurring in the 9 s following reward delivery was related to the US AUC dopamine response (repeated-measures correlation: *r*_rm_ = 0.17, *p *=* *0.02; Extended Data Table 5-1). These results highlight that behavioral responding during early Pavlovian learning is linked to US-evoked dopamine levels and unrelated to CS-evoked dopamine levels.

## Discussion

The dopamine response to cues can signal differences in value-related information in well trained animals (Fiorillo et al., 2003; Tobler et al., 2005; Roesch et al., 2007; Gan et al., 2010; Hart et al., 2015; Fonzi et al., 2017). For example, cue-evoked dopamine release conveys differences in the reward rate (i.e., the time elapsed since the previous reward delivery) after extensive training (Fonzi et al., 2017). However, cue-evoked dopamine release does not signal differences in reward rate during the first six Pavlovian training sessions (Stelly et al., 2021). Our current results extend on these findings and demonstrate that during the first six training sessions, cue-evoked dopamine release did not signal differences in reward size. However, with further training, we find that reward size is encoded by the dopamine response to cues. Together, this suggests that cue-evoked dopamine signals differences in reward value through a multistep process. First, cue-evoked dopamine signals an upcoming reward independent of value. Additional training is then required for cue-evoked dopamine release to encode differences in reward value.

Prior studies have identified sex differences in behavioral responding, as females display elevated motor activity compared with males in locomotor and anxiety-like assays (Scholl et al., 2019; Bishnoi et al., 2021; Knight et al., 2021). Furthermore, sex differences have been observed across a variety of dopamine-dependent behaviors in mice and rats (Zachry et al., 2019; Kutlu et al., 2020; Chen et al., 2021). For example, female rats display a faster acquisition rate and elevated responding during drug self-administration (Lynch and Carroll, 1999; George et al., 2021). Female mice also exhibit higher levels of conditioned freezing relative to males during fear conditioning (Dubroqua et al., 2011). Sex differences have additionally been identified in Pavlovian conditioning tasks using food rewards (Stringfield et al., 2019). Specifically, female rats exhibited greater levels of sign-tracking behavior (e.g., physical interactions with a lever cue), but there were no sex differences in goal-tracking behavior (e.g., head entries to the food receptacle; Stringfield et al., 2019). By using audio cues in our Pavlovian task, animals were not able to engage in standard sign-tracking behaviors. Regardless, we identified prominent sex differences in this Pavlovian task as females exhibited a higher level of goal-tracking compared with males. In addition to behavioral responses occurring during the cue, we analyzed the post-US head entries into the food port. Both males and females performed more head entries following the delivery of the large reward option. Furthermore, female rats performed more head entries compared with males throughout the post-US period. These findings illustrate previously unappreciated sex differences in behavioral responding following the delivery of rewards. We note that the sex differences observed across some of our behavioral metrics (latency and post-US head entries) could be explained by higher levels of motor activity in females (Scholl et al., 2019; Bishnoi et al., 2021; Knight et al., 2021). However, our measure of conditioned responding is normalized to underlining differences in motor activity as we calculate the change in the rate of the head entries during the 5 s CS relative to the rate of head entries during the preceding 5 s. As such the sex differences in conditioned responding in our task cannot be explained solely by increased activity.

We identified stimulus-specific sex differences in dopamine release using fast-scan cyclic voltammetry. Cue-evoked dopamine release did not differ between males and females. In contrast, the reward-evoked dopamine response was lower in females relative to males. Prior research has identified lower levels of dopamine transmission in ovariectomized female rats compared with castrated male rats using microdialysis (Becker and Ramirez, 1981; Castner et al., 1993; Xiao and Becker, 1994). However, one must exercise caution generalizing the findings from ovariectomized females to intact females, as there are no sex differences in basal dopamine levels, as measured by no net flux microdialysis within intact subjects (Xiao and Becker, 1994). Furthermore, a meta-analysis of microdialysis research concludes no basal or drug-induced sex differences in striatal dopamine in rats (Egenrieder et al., 2020). As such, it is unlikely that any potential basal differences in dopamine levels between sexes could account for the stimulus-specific sex differences in rapid dopamine transmission.

Increasing evidence suggests that the estrous stage may contribute to the observed sex differences in dopamine release in mice, rats, and nonhuman primates (Becker and Ramirez, 1981; Castner et al., 1993; Xiao and Becker, 1994; Kritzer and Kohama, 1998; Zhang et al., 2008; Becker et al., 2012; Perez et al., 2014; Calipari et al., 2017; Becker and Chartoff, 2019; Yoest et al., 2019; Zachry et al., 2021). For example, in mice and rats the burst firing rate of dopamine neurons in the ventral tegmental area is elevated during estrus compared with other stages of the cycle in females, as well as compared with males (Perez et al., 2014; Calipari et al., 2017). Additional research finds that female mice elicit higher striatal dopamine release during estrus in response to electrical stimulation and cocaine, as measured using fast-scan cyclic voltammetry in anesthetized subjects (Calipari et al., 2017). We did not monitor the stages of the estrous cycle in the current study, so we cannot assess whether these sex differences in reward-evoked dopamine release are because of cycling hormones. However, the observed sex differences were selective to the reward delivery (and not the cue) and were observed across sessions. Together, this evidence suggests that the sex differences in dopamine transmission are not mediated by the estrous cycle and could instead reflect intrinsic differences between males and females. Additionally, we found no difference in the rate of decay of reward-evoked dopamine release between males and females, which suggests that the observed sex difference is likely not because of differences in dopamine clearance. Future studies are needed to identify the source of these sex differences with *in vivo* dopamine transmission, which may result from anatomic and/or functional differences in the afferent input conveying reward-related information. Recent work identified regional differences in stimulated dopamine release between males and females (Brundage et al., 2021), so it is unclear whether the sex differences in reward-evoked dopamine release are present throughout the striatum and across species. One potential limitation of our study is the lower number of female rats with voltammetry electrodes used. However, we observed robust behavioral effects and changes in dopamine release in females. Regardless, the lower US-evoked dopamine response in females could account for the results from human studies where females exhibit a diminished sensitivity to rewards relative to males (Dhingra et al., 2021).

The magnitude of the dopamine response to the cue presentation and reward delivery have been linked to behavioral outcomes in a variety of Pavlovian conditioning tasks (Darvas et al., 2014; Fonzi et al., 2017; Coddington and Dudman, 2018; Heymann et al., 2020; Lee et al., 2020; Morrens et al., 2020; Stelly et al., 2020, 2021). Our findings demonstrate a positive relationship between head entries and dopamine release following reward delivery, which is likely mediated by the increase in both post-reward head entries and the post-reward dopamine response across training in females. We also observed an inverse correlation between conditioned responding and reward-evoked dopamine release. While this relationship accounts for a relatively small amount of variation in the data, these results parallel our prior research and is consistent with the findings from studies using optogenetic manipulations of the dopamine system (Heymann et al., 2020; Stelly et al., 2020, 2021).

Cue-evoked dopamine release did not encode differences in reward value during the first six training sessions in the current study and in our prior work (Stelly et al., 2021). Our task design used two audio cues, and the rewards were delivered in the same location between trials, which together could dampen the ability of the subject to discriminate between the cues and their corresponding outcomes. It is possible that cue-evoked dopamine release could reflect differences in reward value during earlier training sessions if we had used cues with different sensory modalities. We find that conditioned responding did not differ by reward size and is not related to cue-evoked dopamine release within the first nine training sessions. However, in animals extensively trained under a similar Pavlovian task, an update in cue-evoked dopamine release can elicit a cue-specific change in conditioned responding (Fonzi et al., 2017). Together, these studies indicate that the initial emergence of conditioned responding is linked to the decrease in reward-evoked dopamine release, whereas updates to cue-evoked dopamine release in well trained animals leads to a corresponding update in conditioned responding.

## Synthesis

Reviewing Editor: Laura Bradfield, University of Technology Sydney Decisions are customarily a result of the Reviewing Editor and the peer reviewers coming together and discussing their recommendations until a consensus is reached. When revisions are invited, a fact-based synthesis statement explaining their decision and outlining what is needed to prepare a revision will be listed below. The following reviewer(s) agreed to reveal their identity: Jan Rodriguez Parkitna.

Thank you for submitting your manuscript to eNeuro. The reviewers were generally positive about this manuscript, finding it interesting and well-written. They did raise some minor concerns, as detailed in the specific reviews below.

Reviewer #1

In this study, the authors tracked the behavioral responses and accumbal dopamine release in males and females rats as they acquire Pavlovian approach to two differently valued cues. The study present several interesting findings. First, the study shows that the encoding of expected reward size in dopamine signals emerges relatively late in training (after at least 6 days of training). Second, the study show that, compared to male rats, female rats display an elevated approach behavior (both CS- and US- evoked) and a reduced US-evoked dopamine response. It is clear that the experiments were skillfully executed, the results are clear and appropriately analyzed, and the manuscript is very well written. I have only a few questions/comments, which I hope will strengthen an already excellent manuscript

- Sex differences.

The results of this study are very clear, however the small sample size (8 males and 5 females) somewhat limits the interpretation of this work in terms of broad sex differences (a specific neurobehavioral phenotype could have been accidentally over-represented in one sex). I am not asking authors to add more subjects, but simply to explicitly acknowledge this limitation in the discussion.

- Multi-step encoding of reward value in dopamine neurons.

This study concludes that the encoding of the specific reward value (high vs low reward) in dopamine signals emerges late in training and is secondary to an initial encoding, in early training, of a general upcoming reward (independent of the specific value). While this is clearly the case in this study, it is important to mention that the behavioral paradigm -2 cues of the same modality, followed by the same outcome (identity-wise), delivered in the same location- was not designed to encourage cue discrimination. Indeed, rats show no behavioral evidence of cue discrimination at any stage of training. Therefore, conclusions about the multi-step process of reward encoding in dopamine neurons should be qualified.

- Correlation between post-US entries and post-US dopamine levels.

In the study, females demonstrated elevated post-US approach behavior and reduced post-US dopamine level (AUC). Some reader might assume that this reduced dopamine level corresponds to the accumulation of negative prediction errors computed at each post-US port entry (i.e. visits to empty port). However, the authors report a positive correlation between the number of post-US entries and the post US dopamine levels (probably due to the highest dopamine levels and highest number of port entries after a large reward). This important result should be highlighted to discuss/refute the possible relationship between post-reward dopamine fluctuations (negative AUC) and post-reward negative prediction errors.

On the same topic, can the authors estimate how long it takes for rats to eat 3 pellets (i.e. when post-US responses can be unambiguously classified as empty-port visits)?

Minor points:

Page 4 “Rats were fed ∼15g/day of standard chow”. This would result in different levels of restriction for males and females rats. Can the authors clarify if the quantity of food provided during daily feeding was adjusted for males and females?

Were males and females rats run in the same conditioning chambers in consecutive sessions?

Reviewer #2

The manuscript describes differences between male and female rats in the Pavlovian approach and correlates them with changes in extracellular dopamine levels in the nucleus accumbens assessed using fast-scan cyclic voltammetry. Reported findings extend previously published data, most notably showing considerable sex-dependent differences in the activity of the mesolimbic system during learning.

I would like to note, that due to the fact that Authors have made clearly-marked references to their previous work, the review was not truly “blind”.

My criticisms are minor and mostly relate to the interpretation of the data. In my opinion, some of the methodological aspects of the study may limit the generalization of the observed effects, or suggest potential alternative explanations.

The first is anatomical specificity. Most of the FSCV recordings were taken from the nucleus accumbens core or in its immediate proximity. The Authors discuss the findings in a broader context of dopamine release in the mesolimbic system, without reserving the possibility of differences between nucleus accumbens subareas, in particular the core vs. medial shell.

The second is the model. The male and female rats differed considerably in weight, and I would not discard that as a factor influencing the Pavlovian approach behavior. Were the male and female rats age-matched? (I could not find the exact age of the animals tested)

The third is the species: rats. It remains uncertain whether the observations generalize to other rodents or primates.

In addition, the statistical analysis reported is rigorous and raises no concerns. Nevertheless, I have two comments. First, estimation of standard deviation on small size samples has limited accuracy, thus as a result the rationale for applying a correction to the mixed-model analysis could be arguable. Second, interpretation of a significant correlation with r = -0.15 is limited, a very small amount of variation is explained.

## Author Response

We thank the reviewers for providing thoughtful and constructive commentary. The revised manuscript incorporates the recommended additional discussion points noted by all Reviewers. Our point-by-point response is detailed below.

Synthesis of Reviews:

1. Computational Neuroscience Model Code Accessibility Comments for Author (Required): Im fairly sure this is not applicable, but if the authors did use any custom code (e.g. in Rstudio) please could they include a link to it in the manuscript.

We used the code listed in (Bakdash and Marusich, 2017), which we have referenced in the methods section (line 151).

2. Comments on the Visual Abstract for Author (Required): Reviewer #2 suggested that the visual abstract could be confusing to researchers that dont have a strong behavioural background. Please consider revising. Some suggestions we came up with include: replacing field-specific acronyms such as “US” and “CS” with words such as “cue” and “reward”. Also please be clearer as to what is meant by dopamine in the context of this task.

We have added clarification to the visual abstract, including changing “CS” to “cue", “US” to “reward", and changing “Dopamine” to “Changes in dopamine release”.

Reviewer #1:

1. Sex differences: The results of this study are very clear, however the small sample size (8 males and 5 females) somewhat limits the interpretation of this work in terms of broad sex differences (a specific neurobehavioral phenotype could have been accidentally over-represented in one sex). I am not asking authors to add more subjects, but simply to explicitly acknowledge this limitation in the discussion.

We have added a point of discussion that one potential limitation of our study is the lower number of female rats with voltammetry electrodes used. However we observed robust behavioral effects and changes in dopamine release in females (lines 347-349).

2. Multi-step encoding of reward value in dopamine neurons: This study concludes that the encoding of the specific reward value (high vs low reward) in dopamine signals emerges late in training and is secondary to an initial encoding, in early training, of a general upcoming reward (independent of the specific value). While this is clearly the case in this study, it is important to mention that the behavioral paradigm -2 cues of the same modality, followed by the same outcome (identity-wise), delivered in the same location- was not designed to encourage cue discrimination. Indeed, rats show no behavioral evidence of cue discrimination at any stage of training. Therefore, conclusions about the multi-step process of reward encoding in dopamine neurons should be qualified.

We clarified in the discussion that our task design used two audio cues and the rewards were delivered in the same location between trials, which together could dampen the subjects ability to discriminate between the cues and their corresponding outcomes. It is possible cue-evoked dopamine release could reflect differences in reward value during earlier training sessions if we had employed cues with different sensory modalities. However, we discuss how a change in conditioned responding can be elicited by a change in cue-evoked dopamine release in well-trained animals (see lines 363-372).

3. Correlation between post-US entries and post-US dopamine levels: In the study, females demonstrated elevated post-US approach behavior and reduced post-US dopamine level (AUC). Some reader might assume that this reduced dopamine level corresponds to the accumulation of negative prediction errors computed at each post-US port entry (i.e. visits to empty port). However, the authors report a positive correlation between the number of post-US entries and the post US dopamine levels (probably due to the highest dopamine levels and highest number of port entries after a large reward). This important result should be highlighted to discuss/refute the possible relationship between post-reward dopamine fluctuations (negative AUC) and post-reward negative prediction errors. On the same topic, can the authors estimate how long it takes for rats to eat 3 pellets (i.e. when post-US responses can be unambiguously classified as empty-port visits)?

Our discussion is updated to include the correlation between post-reward head entries and the post-reward dopamine response, which is likely due to the increase in head entries and reward-evoked dopamine release across training in females.

We unfortunately do not have video recordings that are capable of accurately discerning when the food pellets were consumed. However, in the Methods we mention that the food pellets on the Large Reward trials are delivered within the 0.4 s following the CS termination (line 105).

4. Page 4 “Rats were fed ∼15g/day of standard chow”. This would result in different levels of restriction for males and females rats. Can the authors clarify if the quantity of food provided during daily feeding was adjusted for males and females?

Thank you for catching this oversight. We have updated the Methods section to reflect the correct amount of chow given to male or female rats (∼8 g/day for females and ∼15 g/day for males) (line 94).

5. Were males and females rats run in the same conditioning chambers in consecutive sessions?

Male and female rats were run in the same conditioning chambers. To avoid potential transmission of odors, the chamber floors were thoroughly cleaned with a disinfectant, and the walls and food port were cleaned with 70% ethanol solution between every subject. This statement was added to the methods section (lines 97-99).

Reviewer #2:

1. The first is anatomical specificity. Most of the FSCV recordings were taken from the nucleus accumbens core or in its immediate proximity. The Authors discuss the findings in a broader context of dopamine release in the mesolimbic system, without reserving the possibility of differences between nucleus accumbens subareas, in particular the core vs. medial shell.

We have added a discussion point acknowledging recent work illustrating sex differences in stimulated dopamine release across striatal subregions so it is unclear if the sex differences in reward-evoked dopamine release are present throughout the striatum (Brundage et al., 2021) (see lines 344-347).

2. The second is the model. The male and female rats differed considerably in weight, and I would not discard that as a factor influencing the Pavlovian approach behavior. Were the male and female rats age-matched? (I could not find the exact age of the animals tested)

Male and female rats were age-matched to P60-65 days upon arrival. This clarification has been added to the Methods (line 84).

3. The third is the species: rats. It remains uncertain whether the observations generalize to other rodents or primates.

We have clarified throughout the discussion which cited studies utilize rats, mice, or non-human primates for research examining sex differences in dopamine transmission or sex differences in behavioral responding. Furthermore, we note that it is unclear if the sex differences in reward-evoked dopamine release in the current study are present throughout across species (lines 344-347).

4. In addition, the statistical analysis reported is rigorous and raises no concerns. Nevertheless, I have two comments. First, estimation of standard deviation on small size samples has limited accuracy, thus as a result the rationale for applying a correction to the mixed-model analysis could be arguable. Second, interpretation of a significant correlation with r = -0.15 is limited, a very small amount of variation is explained.

While the relationship between conditioned responding and reward-evoked dopamine release accounts for a relatively small amount of variation in the data, we note that these results parallel our prior research and is consistent with the findings from studies employing optogenetic manipulations of the dopamine system (Heymann et al., 2020; Stelly et al., 2020; Stelly et al., 2021) (lines 359-362).

References:

Bakdash JZ, Marusich LR (2017) Repeated Measures Correlation. Front Psychol 8:456.

Brundage JN, Mason CP, Wadsworth HA, Finuf CS, Nelson JJ, Ronstrom PJW, Jones SR, Siciliano CA, Steffensen SC, Yorgason JT (2021) Regional and sex differences in spontaneous striatal dopamine transmission. J Neurochem.

Heymann G, Jo YS, Reichard KL, McFarland N, Chavkin C, Palmiter RD, Soden ME, Zweifel LS (2020) Synergy of Distinct Dopamine Projection Populations in Behavioral Reinforcement. Neuron 105:909-920 e905.

Stelly CE, Tritley SC, Rafati Y, Wanat MJ (2020) Acute Stress Enhances Associative Learning via Dopamine Signaling in the Ventral Lateral Striatum. J Neurosci 40:4391-4400.

Stelly CE, Girven KS, Lefner MJ, Fonzi KM, Wanat MJ (2021) Dopamine release and its control over early Pavlovian learning differs between the NAc core and medial NAc shell. Neuropsychopharmacology.
