# Supplementary material for: Sex Differences in Behavioral Responding and Dopamine Release during Pavlovian Learning
Source: eNeuro. 2022 Mar 21;9(2):ENEURO.0050-22.2022. doi: 10.1523/ENEURO.0050-22.2022 (PMC8941639; doi:10.1523/ENEURO.0050-22.2022)
Supplement: Extended Data Table 1-1 — Panel B - Conditioned responding. Download Table 1-1, DOC file. [file enu-eN-NWR-0050-22-s08.doc]

| Table 1-1 (n = 8 males, n = 5 females) | | | |
| --- | --- | --- | --- |
| Panel B – Conditioned responding | | | |
| Three-way mixed-effects model | Session  *F*_(2.26, 24.86)_ = 14.01, *p* < 0.0001 | Sex  *F*_(1, 55)_ = 3.90, *p* = 0.05 | Reward size  *F*_(1, 11)_ = 0.03, *p* = 0.86 |
| Session x Sex  *F*_(5, 55)_ = 2.34, *p* = 0.05 | Session x Reward size  *F*_(1.91, 20.97)_ = 0.54, *p* = 0.58 | Sex x Reward size  *F*_(1, 55)_ = 0.18, *p* = 0.67 | Three-way interaction  *F*_(5, 55)_ = 1.20, *p* = 0.32 |
| Panel C – Conditioned responding: Sessions 1-3 | | | |
| Two-way mixed-effects model | Reward size  *F*_(1, 11)_ = 0.25, *p* = 0.63 | Sex  *F*_(1, 11)_ = 0.90, *p* = 0.36 | Two-way interaction  *F*_(1, 11)_ = 0.05, *p* = 0.83 |
| Panel D – Conditioned responding: Sessions 4-6 | | | |
| Two-way mixed-effects model | Reward size  *F*_(1, 11)_ = 0.50, *p* = 0.49 | Sex  *F*_(1, 11)_ = 5.11, *p* < 0.05 | Two-way interaction  *F*_(1, 11)_ = 0.54, *p* = 0.48 |
| Panel E – Latency to respond | | | |
| Three-way mixed-effects model | Session  *F*_(2.94, 32.33)_ = 6.26, *p* < 0.002 | Sex  *F*_(1, 55)_ = 8.80, *p* = 0.004 | Reward size  *F*_(1, 11)_ = 0.51, *p* = 0.49 |
| Session x Sex  *F*_(5, 55)_ = 0.60, *p* = 0.70 | Session x Reward size  *F*_(2.12, 23.32)_ = 1.40, *p* = 0.27 | Sex x Reward size  *F*_(1, 55)_ = 0.72, *p* = 0.40 | Three-way interaction  *F*_(5, 55)_ = 2.40, *p* < 0.05 |
| Panel F – Latency to respond: Sessions 1-3 | | | |
| Two-way mixed-effects model | Reward size  *F*_(1, 11)_ = 1.71, *p* = 0.22 | Sex  *F*_(1, 11)_ = 14.56, *p* = 0.003 | Two-way interaction  *F*_(1, 11)_ = 1.25, *p* = 0.29 |
| Panel F – Latency to respond: Sessions 4-6 | | | |
| Two-way mixed-effects model | Reward size  *F*_(1, 11)_ = 0.01, *p* = 0.94 | Sex  *F*_(1, 11)_ = 2.71, *p* = 0.13 | Two-way interaction  *F*_(1, 11)_ = 0.15, *p* = 0.71 |
