# Supplementary material for: Sex Differences in Behavioral Responding and Dopamine Release during Pavlovian Learning
Source: eNeuro. 2022 Mar 21;9(2):ENEURO.0050-22.2022. doi: 10.1523/ENEURO.0050-22.2022 (PMC8941639; doi:10.1523/ENEURO.0050-22.2022)
Supplement: Extended Data Table 2-1 — Panel B -Post US head entries. Download Table 2-1, DOC file. [file enu-eN-NWR-0050-22-s09.doc]

| Table 2-1 | | | | |
| --- | --- | --- | --- | --- |
| Panel B –Post US head entries | | | | |
| Three-way mixed-effects model | Session  *F*_(1.98, 21.81)_ = 1.30, *p* = 0.29 | Sex  *F*_(1, 55)_ = 17.44, *p* = 0.0001 | | Reward size  *F*_(1, 11)_ = 10.15, *p* = 0.009 |
| Session x Sex  *F*_(5, 55)_ = 1.79, *p* = 0.13 | Session x Reward size  *F*_(2.52, 27.69)_ = 1.62, *p* = 0.21 | Sex x Reward size  *F*_(1, 55)_ = 1.77, *p* = 0.19 | | Three-way interaction  *F*_(5, 55)_ = 0.94, *p* = 0.46 |
| Panel C – Early US head entries | | | | |
| Three-way mixed-effects model | Session  *F*_(2.49, 27.40)_ = 1.72, *p* = 0.19 | | Sex  *F*_(1, 55)_ = 13.60, *p* = 0.0005 | Reward size  *F*_(1, 11)_ = 1.45, *p* = 0.25 |
| Session x Sex  *F*_(5, 55)_ = 0.99, *p* = 0.43 | Session x Reward size  *F*_(3.02, 33.24)_ = 0.68, *p* = 0.57 | | Sex x Reward size  *F*_(1, 55)_ = 1.54, *p* = 0.22 | Three-way interaction  *F*_(5, 55)_ = 0.93, *p* = 0.47 |
| Panel D – Late US head entries | | | | |
| Three-way mixed-effects model | Session  *F*_(1.65, 18.10)_ = 0.67, *p* = 0.50 | Sex  *F*_(1, 55)_ = 12.20, *p* = 0.001 | | Reward size  *F*_(1, 11)_ = 24.65, *p* = 0.0004 |
| Session x Sex  *F*_(5, 55)_ = 1.83, *p* = 0.12 | Session x Reward size  *F*_(2.01, 22.15)_ = 2.92, *p* = 0.07 | Sex x Reward size  *F*_(1, 55)_ = 5.94, *p* = 0.02 | | Three-way interaction  *F*_(5, 55)_ = 1.50, *p* = 0.21 |
