# Supplementary material for: Sex Differences in Behavioral Responding and Dopamine Release during Pavlovian Learning
Source: eNeuro. 2022 Mar 21;9(2):ENEURO.0050-22.2022. doi: 10.1523/ENEURO.0050-22.2022 (PMC8941639; doi:10.1523/ENEURO.0050-22.2022)
Supplement: Extended Data Table 3-1 — Panel D - CS-evoked dopamine release. Download Table 3-1, DOC file. [file enu-eN-NWR-0050-22-s10.doc]

| Table 3-1 (males: n = 9 electrodes; females: n = 5 electrodes) | | | |
| --- | --- | --- | --- |
| Panel D – CS-evoked dopamine release | | | |
| Three-way mixed-effects model | Session  *F*_(1.97, 23.64)_ = 3.22, *p =* 0.06 | Sex  *F*_(1, 30)_ = 0.07, *p* = 0.80 | Reward size  *F*_(1, 12)_ = 3.54, *p* = 0.09 |
| Session x Sex  *F*_(5, 30)_ = 2.48, *p* = 0.05 | Session x Reward size  *F*_(2.78, 16.70)_ = 2.78, *p* = 0.08 | Sex x Reward size  *F*_(1, 30)_ = 0.19, *p* = 0.67 | Three-way interaction  *F*_(5, 30)_ = 0.63, *p* = 0.68 |
| Panel E – Peak US-evoked dopamine release | | | |
| Three-way mixed-effects model | Session  *F*_(2.43, 29.14)_ = 13.18, *p* < 0.0001 | Sex  *F*_(1, 30)_ = 3.14, *p* = 0.09 | Reward size  *F*_(1, 12)_ = 17.40, *p =* 0.001 |
| Session x Sex  *F*_(5, 30)_ = 0.47, *p* = 0.80 | Session x Reward size  *F*_(1.36, 8.14)_ = 0.27, *p* = 0.69 | Sex x Reward size  *F*_(1, 30)_ = 1.97, *p* = 0.17 | Three-way interaction  *F*_(5, 30)_ = 1.73, *p* = 0.16 |
| Panel F – AUC US-evoked dopamine release | | | |
| Three-way mixed-effects model | Session  *F*_(2.06, 24.72)_ = 2.54, *p* = 0.10 | Sex  *F*_(1, 30)_ = 7.91, *p* = 0.009 | Reward size  *F*_(1, 12)_ = 17.98, *p* = 0.001 |
| Session x Sex  *F*_(5, 30)_ = 6.56, *p* = 0.0003 | Session x Reward size  *F*_(1.96, 11.74)_ = 0.65, *p* = 0.54 | Sex x Reward size  *F*_(1, 30)_ = 0.18, *p* = 0.67 | Three-way interaction  *F*_(5, 30)_ = 1.36, *p* = 0.27 |
