# Supplementary material for: Sex Differences in Behavioral Responding and Dopamine Release during Pavlovian Learning
Source: eNeuro. 2022 Mar 21;9(2):ENEURO.0050-22.2022. doi: 10.1523/ENEURO.0050-22.2022 (PMC8941639; doi:10.1523/ENEURO.0050-22.2022)
Supplement: Extended Data Table 4-1 — Panel B - CS-evoked dopamine: Sessions 7-9. Download Table 4-1, DOC file. [file enu-eN-NWR-0050-22-s11.doc]

| Table 4-1 | | | |
| --- | --- | --- | --- |
| Panel B – CS-evoked dopamine: Sessions 7-9 | | | |
| Two-way mixed-effects model | Reward size  *F*_(1, 10)_ = 5.78, *p* = 0.04 | Sex  *F*_(1, 10)_ = 0.16, *p* = 0.70 | Two-way interaction  *F*_(1, 10)_ = 0.001, *p* = 0.97 |
| Panel C – Peak US dopamine: Sessions 7-9 | | | |
| Two-way mixed-effects model | Reward size  *F*_(1, 10)_ = 2.83, *p* = 0.12 | Sex  *F*_(1, 10)_ = 1.74, *p* = 0.22 | Two-way interaction  *F*_(1, 10)_ = 1.68, *p* = 0.22 |
| Panel D – AUC US dopamine: Sessions 7-9 | | | |
| Two-way mixed-effects model | Reward size  *F*_(1, 10)_ = 24.54, *p* = 0.0006 | Sex  *F*_(1, 10)_ = 8.76, *p* = 0.01 | Two-way interaction  *F*_(1, 10)_ = 0.03, *p* = 0.87 |
