# Supplementary material for: Sex Differences in Behavioral Responding and Dopamine Release during Pavlovian Learning
Source: eNeuro. 2022 Mar 21;9(2):ENEURO.0050-22.2022. doi: 10.1523/ENEURO.0050-22.2022 (PMC8941639; doi:10.1523/ENEURO.0050-22.2022)
Supplement: Extended Data Table 5-1 — Sessions 1-9 Repeated measures correlation. Download Table 5-1, DOC file. [file enu-eN-NWR-0050-22-s12.doc]

| Table 5-1 | |
| --- | --- |
| Sessions 1-9 Repeated measures correlation | |
| Panel A – CS dopamine vs conditioned responding | r_rm_ = -0.04, *p* = 0.60 |
| Panel B – peak US dopamine vs conditioned responding | r_rm_ = -0.15, *p* = 0.04 |
| Additional analyses | |
| CS dopamine vs latency to respond | r_rm_ = 0.06, *p* = 0.40 |
| AUC US dopamine vs 9s post-US HE | r_rm_ = 0.17, *p* = 0.02 |
| AUC US dopamine vs conditioned responding | r_rm_ = -0.02, *p* = 0.82 |
