# Supplementary figures and images for: Sex Differences in Behavioral Responding and Dopamine Release during Pavlovian Learning
Source: eNeuro. 2022 Mar 21;9(2):ENEURO.0050-22.2022. doi: 10.1523/ENEURO.0050-22.2022 (PMC8941639; doi:10.1523/ENEURO.0050-22.2022)

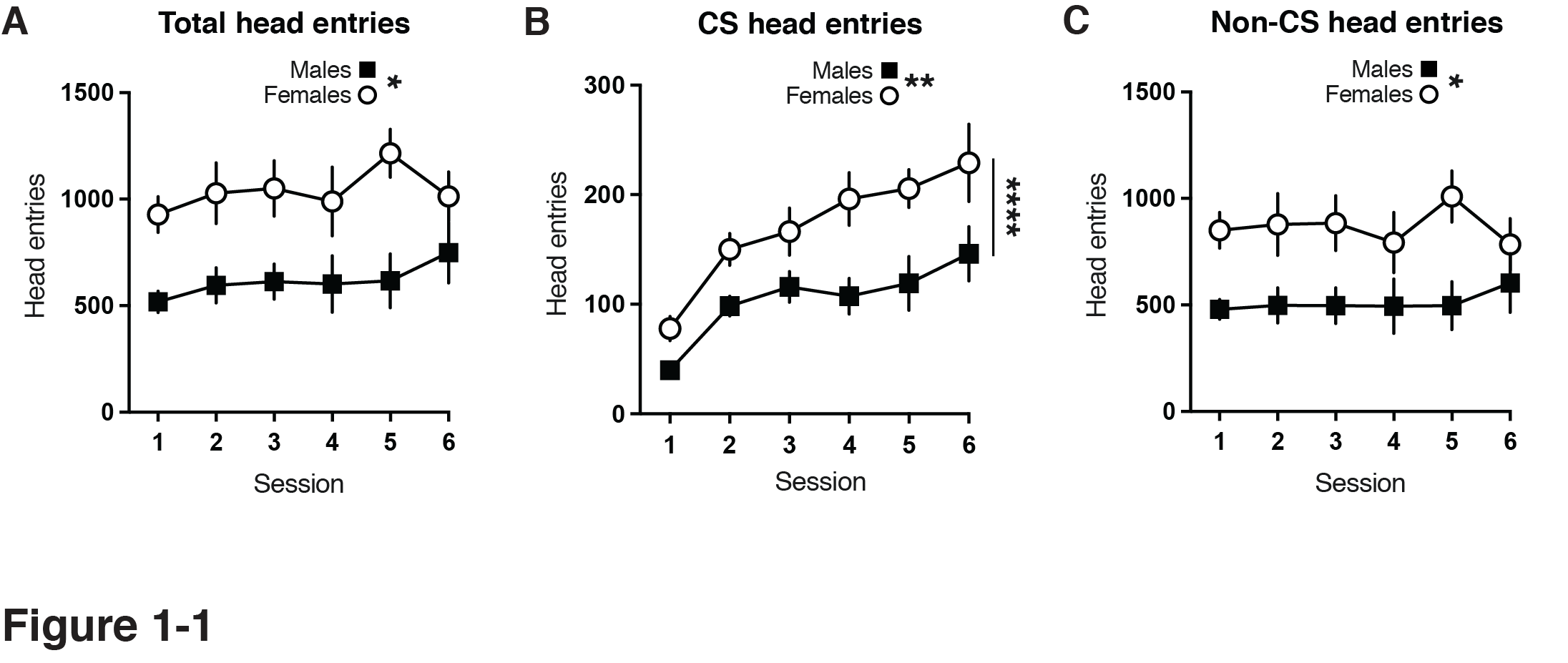

Supplement: Figure 1-1 — Number of head entries across sessions. A, Total number of head entries across sessions in males (black square) and females (open circle; two-way mixed-effects analysis; session effect: F(5,55) = 1.98, p = 0.10; sex effect: F(1,11) = 8.38, p = 0.02; interaction effect: F(5,55) = 1.25, p = 0.30). B, CS head entries across sessions (two-way mixed-effects analysis; session effect: F(2.25,26.69) = 15.34, p < 0.0001; sex effect: F(1,11) = 12.10, p = 0.005; interaction effect: F(5,55) = 0.97, p = 0.44). C, Non-CS head entries across sessions (two-way mixed-effects analysis; session effect: F(2.47,27.11) = 0.73, p = 0.52; sex effect: F(1,11) = 6.12, p = 0.03; interaction effect: F(5,55) = 1.59, p = 0.18). *p < 0.05, **p < 0.01. Download Figure 1-1, TIF file. [file enu-eN-NWR-0050-22-s03.tif]

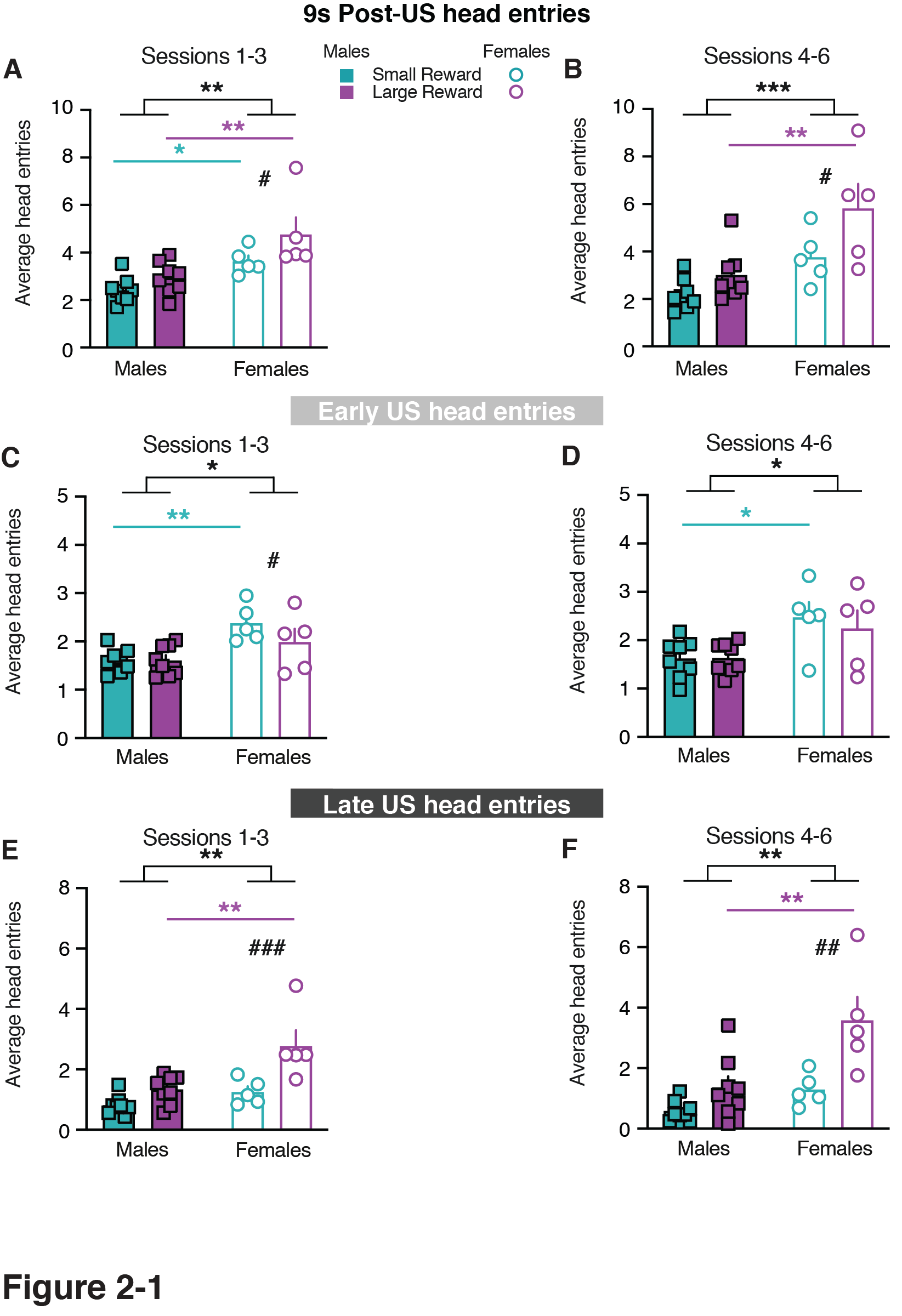

Supplement: Figure 2-1 — Sex differences in behavioral responding during US presentation. A, Head entries during the 9 s postreward window averaged across the first three sessions of training (two-way mixed-effects analysis; reward size effect: F(1,11) = 15.03, p = 0.003; sex effect: F(1,11) = 11.27, p = 0.006; interaction effect: F(1,11) = 2.17, p = 0.17; post hoc Sidak’s test—sex: Small Reward: t(22) = 2.41, p < 0.05; Large Reward: t(22) = 3.67, p = 0.003; post hoc Sidak’s test—reward size: males: t(11) = 1.94, p = 0.15; females: t(11) = 3.41, p = 0.01). B, Head entries during the 9 s postreward window averaged across the latter three sessions of training (two-way mixed-effects analysis; reward size effect: F(1,22) = 7.48, p = 0.01; sex effect: F(1,22) = 17.45, p = 0.0004; interaction effect: F(1,22) = 1.44, p = 0.24; post hoc Sidak’s test—sex; Small Reward: t(22) = 2.11, p = 0.09; Large Reward: t(22) = 3.80, p = 0.002; post hoc Sidak’s test—reward size; males: t(22) = 1.24, p = 0.41; females: t(22) = 2.51, p = 0.04). C, Head entries during the Early US averaged across the first three sessions of training (two-way mixed-effects analysis; reward size effect: F(1,11) = 5.47, p = 0.04; sex effect: F(1,11) = 8.45, p = 0.01; interaction effect: F(1,11) = 5.29, p = 0.04; post hoc Sidak’s test—sex: Small Reward: t(22) = 3.57, p = 0.003; Large Reward: t(22) = 1.78, p = 0.17; post hoc Sidak’s test—reward size; males: t(11) = 0.03, p = 0.99; females: t(11) = 2.96, p = 0.03). D, Head entries during the Early US averaged across the latter three sessions of training (two-way mixed-effects analysis; reward size effect: F(1,11) = 0.27, p = 0.61; sex effect: F(1,11) = 9.67, p = 0.01; interaction effect: F(1,11) = 0.35, p = 0.57; post hoc Sidak’s test—sex; Small Reward: t(22) = 2.72, p = 0.02; Large Reward: t(22) = 1.94, p = 0.13; post hoc Sidak’s test—reward size; males: t(11) = 0.71, p = 0.99; females: t(11) = 0.71, p = 0.74). E, Head entries during the Late US averaged across the first three sessions o [file enu-eN-NWR-0050-22-s04.tif]

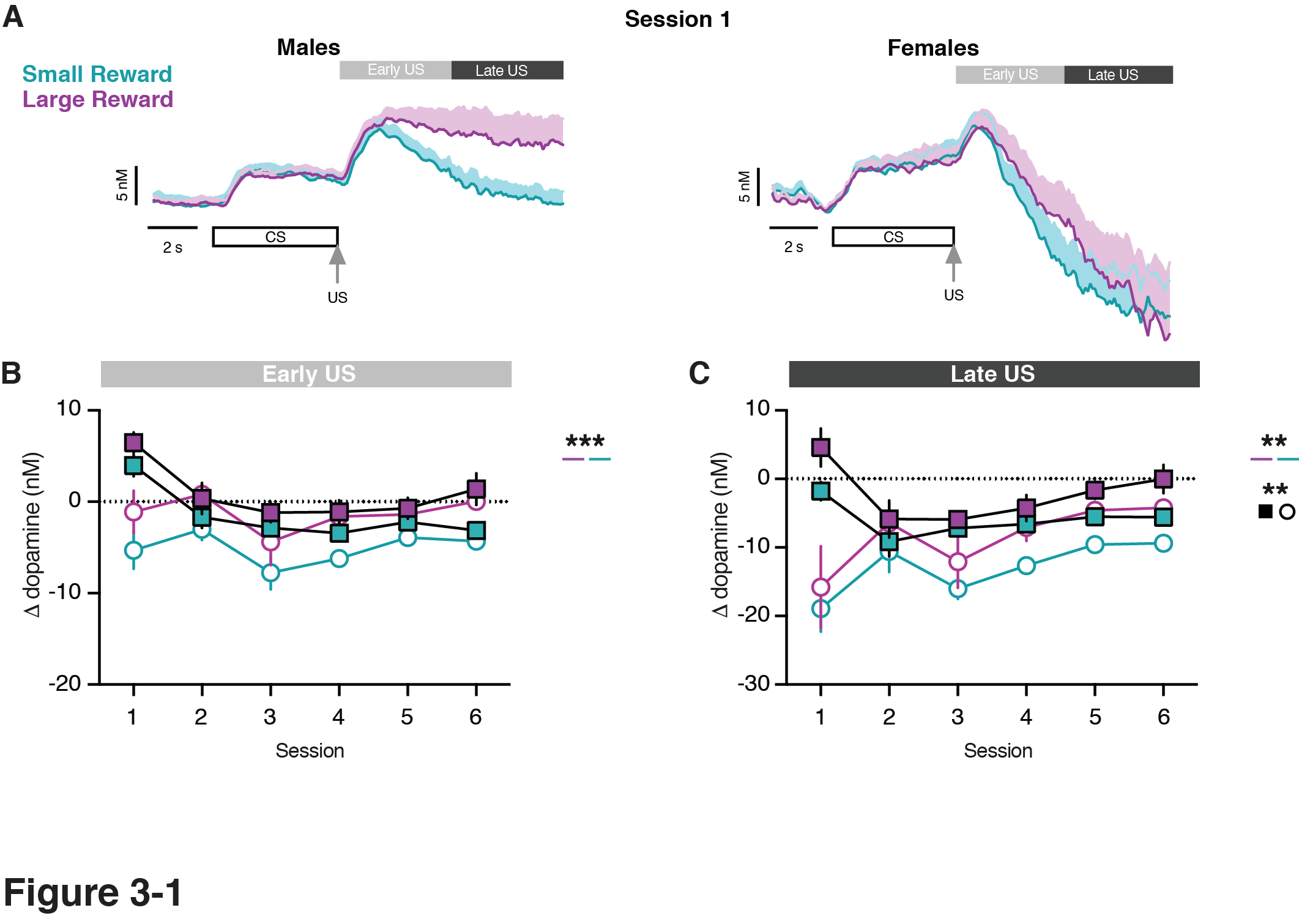

Supplement: Figure 3-1 — Post-US dopamine response in Early and Late epochs. A, Average dopamine signals in first session in males (left) and females (right) depicting Early and Late epochs. B, Average Early US-evoked dopamine release across sessions (three-way mixed-effects analysis; session effect: F(2.77,33.19) = 8.43, p = 0.0004; sex effect: F(1,30) = 3.68, p = 0.06; reward size effect: F(1,12) = 19.10, p = 0.0009; session × sex effect: F(5,30) = 4.64, p = 0.003; session × reward size effect: F(2.38,14.28) = 1.18, p = 0.34; sex × reward size effect: F(1,30) = 1.01, p = 0.32; interaction effect: F(5,30) = 1.22, p = 0.33). C, Average Late US-evoked dopamine release across sessions (three-way mixed-effects analysis; session effect: F(1.80,21.56) = 2.04, p = 0.16; sex effect: F(1,30) = 9.49, p = 0.004; reward size effect: F(1,12) = 15.32, p = 0.002; session × sex effect: F(5,30) = 6.46, p = 0.0003; session × reward size effect: F(1.77,10.62) = 0.58, p = 0.56; sex × reward size effect: F(1,30) = 0.01, p = 0.93; interaction effect: F(5,30) = 1.62, p = 0.18). **p < 0.01, ***p < 0.001. Download Figure 3-1, TIF file. [file enu-eN-NWR-0050-22-s05.tif]

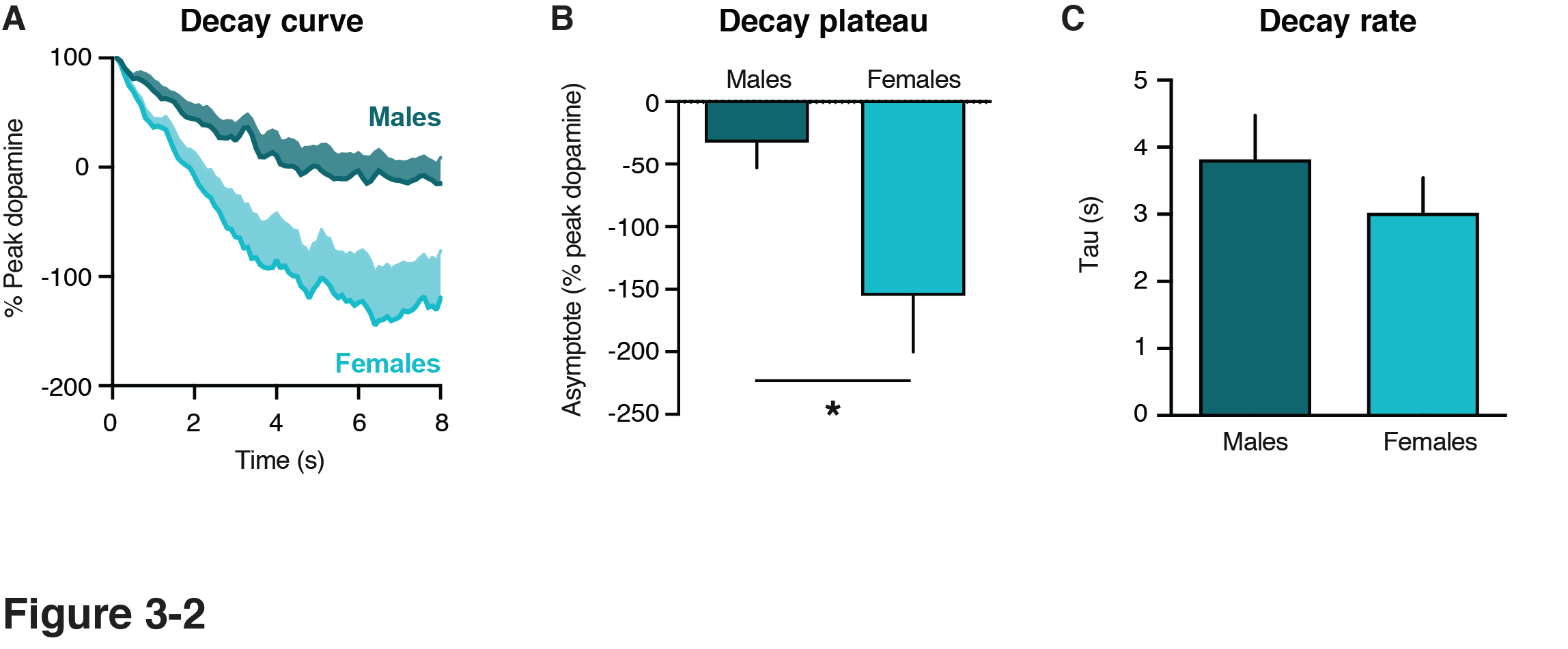

Supplement: Figure 3-2 — Single-phase decay analysis on US dopamine response. A, Average US-evoked dopamine signals normalized to the peak dopamine response in Small Reward trials during the first session in males and females. B, Decay plateau (unpaired t test; t(9) = 2.65, p = 0.03). C, Decay rate (unpaired t test; t(9) = 0.92, p = 0.38). *p < 0.05. Download Figure 3-2, TIF file. [file enu-eN-NWR-0050-22-s06.tif]

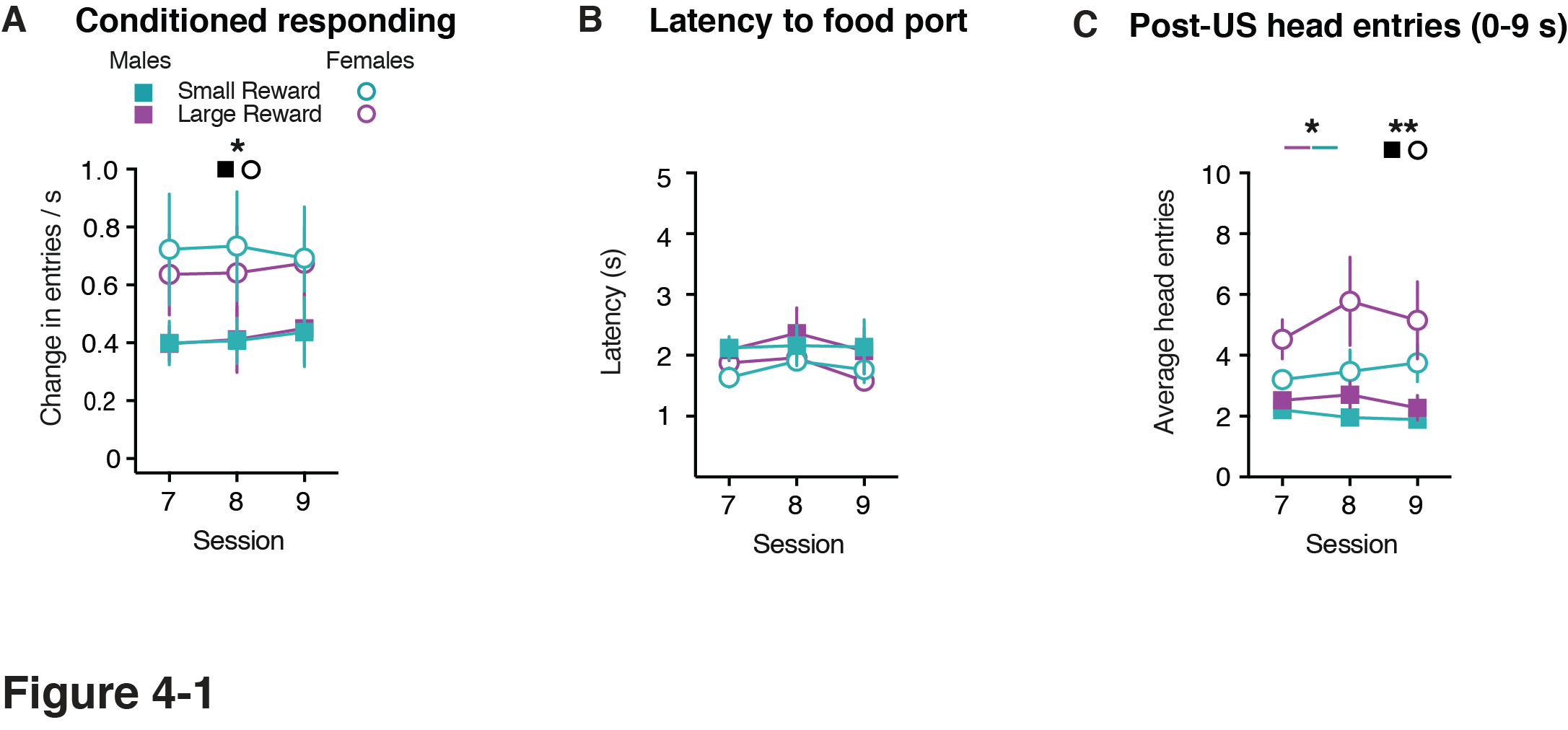

Supplement: Figure 4-1 — Behavioral responding during sessions 7–9. A, Conditioned responding (three-way mixed-effects analysis; session effect: F(1.99,21.95) = 0.42, p = 0.66; sex effect: F(1,18) = 5.56, p = 0.03; reward size effect: F(1,11) = 0.11 p = 0.74; session × sex effect: F(2,18) = 0.33, p = 0.72; session × reward size effect: F(1.76,15.87) = 0.23, p = 0.77; sex × reward size effect: F(1,18) = 0.13, p = 0.72; interaction effect: F(2,18) = 0.13, p = 0.88). B, Latency to respond (three-way mixed-effects analysis; session effect: F(1.68,18.46) = 1.00, p = 0.37; sex effect: F(1,11) = 1.68, p = 0.84; reward size effect: F(1,11) = 0.04 p = 0.84; session × sex effect: F(2,18) = 0.07, p = 0.94; session × reward size effect: F(1.83,16.43) = 1.61, p = 0.23; sex × reward size effect: F(1,18) = 0.003, p = 0.95; interaction effect: F(2,18) = 1.51, p = 0.25). C, Post-US head entries (three-way mixed-effects analysis; session effect: F(1.52,16.67) = 0.86, p = 0.41; sex effect: F(1,18) = 14.62, p = 0.001; reward size effect: F(1,11) = 5.82 p = 0.03; session × sex effect: F(2,18) = 1.46, p = 0.26; session × reward size effect: F(1.25,11.20) = 0.93, p = 0.38; sex × reward size effect: F(1,18) = 1.93, p = 0.18; interaction effect: F(2,18) = 0.17, p = 0.84). *p < 0.05, **p < 0.01. Download Figure 4-1, TIF file. [file enu-eN-NWR-0050-22-s07.tif]
